# The single cyclic nucleotide-specific phosphodiesterase of the intestinal parasite *Giardia lamblia* represents a potential drug target

**DOI:** 10.1371/journal.pntd.0005891

**Published:** 2017-09-15

**Authors:** Stefan Kunz, Vreni Balmer, Geert Jan Sterk, Michael P. Pollastri, Rob Leurs, Norbert Müller, Andrew Hemphill, Cornelia Spycher

**Affiliations:** 1 Institute of Parasitology, Vetsuisse Faculty, University of Bern, Bern, Switzerland; 2 Division of Medicinal Chemistry, Faculty of Sciences, Amsterdam Institute of Molecules, Medicines and Systems (AIMMS), Vrije Universiteit Amsterdam, Amsterdam, The Netherlands; 3 Department of Chemistry and Chemical Biology, Northeastern University, Boston, Massachusetts, United States of America; University of Melbourne, AUSTRALIA

## Abstract

**Background:**

Giardiasis is an intestinal infection correlated with poverty and poor drinking water quality, and treatment options are limited. According to the Center for Disease Control and Prevention, *Giardia* infections afflict nearly 33% of people in developing countries, and 2% of the adult population in the developed world. This study describes the single cyclic nucleotide-specific phosphodiesterase (PDE) of *G*. *lamblia* and assesses PDE inhibitors as a new generation of anti-giardial drugs.

**Methods:**

An extensive search of the *Giardia* genome database identified a single gene coding for a class I PDE, GlPDE. The predicted protein sequence was analyzed *in-silico* to characterize its domain structure and catalytic domain. Enzymatic activity of GlPDE was established by complementation of a PDE-deficient *Saccharomyces cerevisiae* strain, and enzyme kinetics were characterized in soluble yeast lysates. The potency of known PDE inhibitors was tested against the activity of recombinant GlPDE expressed in yeast and against proliferating *Giardia* trophozoites. Finally, the localization of epitope-tagged and ectopically expressed GlPDE in *Giardia* cells was investigated.

**Results:**

*Giardia* encodes a class I PDE. Catalytically important residues are fully conserved between GlPDE and human PDEs, but sequence differences between their catalytic domains suggest that designing *Giardia*-specific inhibitors is feasible. Recombinant GlPDE hydrolyzes cAMP with a K_m_ of 408 μM, and cGMP is not accepted as a substrate. A number of drugs exhibit a high degree of correlation between their potency against the recombinant enzyme and their inhibition of trophozoite proliferation in culture. Epitope-tagged GlPDE localizes as dots in a pattern reminiscent of mitosomes and to the perinuclear region in *Giardia*.

**Conclusions:**

Our data strongly suggest that inhibition of *G*. *lamblia* PDE activity leads to a profound inhibition of parasite proliferation and that GlPDE is a promising target for developing novel anti-giardial drugs.

## Author summary

Cellular signaling by the cyclic nucleotides cAMP and cGMP is ubiquitously found in organisms from human to unicellular parasites. Cyclic nucleotide-specific phosphodiesterases (PDEs) are pivotal regulators of these signaling processes and these enzymes represent important drug targets for a variety of diseases. Eleven PDE families are distinguished in humans and selective inhibition of a single human PDE family without targeting others is feasible. In parasites, interference in the signaling mechanism by PDE inhibition may be fatal. The diarrhea-causing parasite *Giardia lamblia* contains only one single PDE, named GlPDE. GlPDE activity is highly impaired by a range of PDE inhibitors, which also suppress parasite proliferation *in vitro*. Thus, there is a good agreement between PDE inhibition and parasite drug susceptibility. We demonstrate molecular differences between human PDEs and GlPDE that can be exploited for the development of GlPDE-selective inhibitors. Finally, our data may suggest localization of GlPDE to mitosome organelles, which are absent in human cells and thus are in the focus as possible targets for the treatment of giardiasis. This may add to the notion that GlPDE represents a potential target for the development of novel anti-giardial drugs.

## Introduction

*Giardia lamblia* is a protozoan parasite that causes giardiasis, an intestinal disease with symptoms such as diarrhea, nausea, and malabsorption [1]. Trophozoites are the disease-causing stage and colonize the upper small intestine of humans and other vertebrates. They form cysts, which are shed into the environment via the fecal route and which are then orally transmitted, mostly via contaminated water. Giardiasis occurs worldwide, predominantly in resource-poor countries with low standards of sanitation, and represents a major cause of non-bacterial diarrhea with 280 million symptomatic human cases every year [2]. In developing countries, infection rates of 10% to 30% are common, though rates of 40% and higher have been reported in some instances [3,4]. Chronic or recurrent giardiasis in early childhood is associated with poor cognitive function and failure to thrive [5].

Metronidazole (commercially known as Flagyl) and other nitroimidazoles are being used as a therapy of choice since the 1960s. However, resistance against metronidazole has been described [6,7]. Consequently, substitution therapies including the benzimidazole albendazole, the acridine derivative quinacrine—or the aminoglycoside paromomycin, alone or in combination with metronidazole [8,9], are of increasing importance. New therapies are urgently needed because current treatments (i) depend on repeated dosing schedules (suboptimal for developing countries), (ii) have adverse effects, (iii) are ineffective in up to 20% of cases and (iv) clinical or laboratory-induced resistance has been reported for most of the current anti-giardial drugs [10,11].

Phosphodiesterases (PDEs) are key enzymes of cyclic nucleotide signaling. They constitute the only enzymes for hydrolyzing the signaling molecules cAMP and cGMP and thus are crucially important regulators of the temporal and spatial shape of the cyclic nucleotide signals. Three structurally distinct classes of PDEs have been described [12]. Thereof only class I enzymes have been identified in protozoan parasites and their mammalian hosts so far. Human PDEs (hPDEs) comprise eleven class I families (hPDE1–11), which differ with respect to substrate-specificity, regulation and distribution in tissues as well as in intracellular compartments. The catalytic domains of class I PDEs are highly conserved at the level of their three-dimensional structures, though the different families share only 20–50% amino acid sequence identity within their catalytic domains (S1 Table). Small differences in structure and sequence of their catalytic pockets account for substrate selectivity (cAMP versus cGMP) and—most importantly—have allowed the development of family-specific PDE inhibitors [13]. Most hPDE families are being actively studied as potential drug targets against a wide range of medical conditions and a number of PDE inhibitors are currently marketed for various conditions such as chronic obstructive pulmonary disease, psoriatic arthritis or erectile dysfunction [14,15].

The extensive available knowledge on PDE structure, physiology and pharmacology has prompted the study of PDEs as potential targets for the treatment of infectious diseases. In *Trypanosoma brucei*, the causative agent of African sleeping sickness, targeting PDEs by genetic or pharmacological means led to rapid parasite death *in vitro* and to the elimination of infections *in vivo* [16]. Two inhibitors developed against the trypanosomal PDEs TbrPDEB1 and TbrPDEB2, namely NPD-001 and VUF13525, exhibit potencies in the nanomolar range [17,18]. Recent studies in the malaria parasite *Plasmodium falciparum* have demonstrated that a PDE-inhibitor with specificity to a PDE expressed in the merozoite stage of the parasite led to the premature egress of merozoites, thus interrupting the multiple cycles of reinvasion and multiplication [19]. In addition, genetic deletion of PDEγ in the rodent malaria pathogen *Plasmodium yoelii* blocked sporozoite motility and parasite transmission [20]. In *Giardia*, cAMP has been suggested to be involved in excystation [21] and encystation [22], and a potential role in regulating trophozoite attachment and locomotion was hypothesized based on the intracellular localization of the protein kinase A (gPKA) [21]. Thus, PDEs could play a vital role in the biology of this parasite.

In this study, we show that the repertoire of PDEs in the genome of the intestinal parasite *G*. *lamblia* is restricted to a single gene coding for a class I PDE (GlPDE). We expressed GlPDE lacking its N-terminal transmembrane helix region in yeast and characterized its enzymatic activity. We also show that the inhibition profile of a set of well-characterized inhibitors for human PDEs shows a good correlation between enzymatic GlPDE inhibition and anti-parasitic activity, and we have indication that GlPDE is associated with mitosomes, which are relic organelles in *G*. *lamblia*. Our results suggest that GlPDE represents a novel, and potentially promising drug target that should be further exploited for the development of novel and urgently needed medication against giardiasis.

## Materials and methods

### Materials

Resazurin sodium salt (cat. R7017), dimethyl sulfoxide (DMSO, cat. D8418) as well as cAMP and cGMP (sodium salts) were obtained from Sigma (Buchs, Switzerland). Radiochemicals were from Hartmann Analytic (Braunschweig, Germany). PDE inhibitors were from the following sources: isobutyl-methyl-xanthine (IBMX), 8-methoxymethyl-IBMX, vinpocetine, rolipram, dipyridamole, papaverine, BAY 73–6691, BRL 50481 were from Sigma; zardaverine, cilostamide were from BioMol Anawa Trading (Wangen, Switzerland); pentoxifylline was from Calbiochem (Merck Millipore, Schaffhausen, Switzerland); etatolate was from Tocris Bioscience (Lucerna-Chem, Lucerne, Switzerland); erythro-9-(2-hydroxy-3-nonyl)adenine (EHNA), milrinone, trequinsin, Ro 20–1724, dipyridamole, zaprinast were obtained from Fisher Scientific; BAY 60–7550 was from Biotrend Chemicals (Destin, FL, USA), sildenafil citrate was generously provided by Pfizer, Inc. The following compounds were synthesized using methods previously described: NEU28 (syn. PQ-10, CAS 927691-21-2) [23], NEU222 [24], PFE-PDE1 (patent EP911333A1) [24], PFE-PDE9 (patent WO2003037899A1) [24], tadalafil [25], piclamilast (patent WO9212961), roflumilast (patent WO9501338), NPD-001 (syn. CpdA, PPS54019) [26,17] and VUF13525 (compound 20b in Orrling KM et al (2012) [18]).

### *In silico* sequence analyses

The HMMER software package (version 3.1b1) was used to search the GiardiaDB databases (v 4.0) of annotated proteins and translated open reading frames (ORFs) for the presence of PDEs belonging to either of the currently established three PDE classes I, II and III [27]. The profile hidden Markov models (HMMs) were generated from multiple sequence alignments of each PDE class: The HMM of class I PDEs was built from 24 catalytic domain sequences comprising one member of all 11 human PDE families, PDEs from kinetoplastid and plasmodial parasites, as well as PDEs from *Drosophila melanogaster*, *Caenorhabditis elegans* and *Saccharomyces cerevisiae*. The HMM of class II PDEs is based on all 7 manually annotated sequences in the Swiss-Prot database (release2014-04; entries CPDP_ALIFS, CPDP_YERPE, PDE1_CANAX, PDE1_DICDI, PDE1_SCHPO, PDE1_YEAST, PDE7_DICDI). To identify potential class III PDEs, two HMMs were created: (a) by using the twenty most diverse members of the NCBI CDD family PRK11148 (http://www.ncbi.nlm.nih.gov/cdd); (b) by using a seed alignment of 49 sequences contained in the HAMAP profile MF_00905 [28]. All hits above the significance threshold (e < 0.0001) were analyzed manually.

Sequence patterns searches were done with the programs fuzzpro and patmatdb (EMBOSS software package, http://emboss.open-bio.org). Used consensus search patterns were: H-D-[LIVMFY]-x-H-x-[AG]-x(2)-[NQ]-x-[LIVMFY] (class I PDEs, Prosite PS00126), H-x-H-L-D-H-[LIVM]-x-[GS]-[LIVMA]-[LIVM](2)-x-S-[AP] (class II PDEs, Prosite PS00607), D-x(1,1000)-G-D-x(1,1000)-G-N-H-[ED]-x(1,1000)-H-x(1,1000)-G-H-x-H (extended dimetallophosphoesterase pattern).

Multiple sequence alignments (MSAs) were created using MSAprobs [29] (profile-based algorithm) and Expresso [30] (structure-guided algorithm) and then manually refined by incorporating information of solved PDE structures. Conservation scoring analysis of MSAs was done using valdar01 and trident scoring algorithms [31]. Identity scores were calculated using a self-written Python script. Transmembrane helices were predicted using the programs TOPCONS 2.0 [32] (metasearch with five programs), PSIPRED [33] (MEMSAT-SVM and MEMSAT3 algorithms), Phobius/PolyPhobius [34], TMHMM 2.0 [35], TMSEG, PHDhtm [36], TMPred [37] and TopPred [38], and a consensus was deduced from all predictions. Secondary structure predictions were done using iTasser, PSSpred and the “Predict Protein” web services [39,36].

### *Giardia lamblia* cell culture

*G*. *lamblia* WBC6 (ATCC catalogue number 50803) trophozoites were grown under microaerophilic conditions in 11 ml culture tubes (Nunc, cat. 156758) containing TYI-S- 33 medium supplemented with 10% bovine serum and bovine bile according to standard protocols [40]. Parasites were harvested by chilling the tubes on ice for 30 minutes to detach adherent cells and collected by centrifugation (900 x g, 10 minutes, 4°C). Encystation was induced using the two-step method as described previously [41] by cultivating the trophozoites in bile-free medium for 44 hours and thereafter in medium (pH 7.85) containing porcine bile.

### Expression in yeast

The DNA sequence comprising the catalytic domain (residues Gly-983 –Glu-1371) was amplified by PCR using genomic DNA of *G*. *lamblia* WBC6 and the primer pair GlPDE1cat-for (5'-GCA*GTCGA****C*ATATG**GGAGTGGATTGTTCAAATATCAAGTTGG-3') and GlPDE1cat-rev (5'-CGTGGATCCTATTACTCCTTCTTGTCCAGCTCAATCTC-3'). The PCR product was then cloned into the TA vector pCRII-TOPO (Invitrogen) yielding plasmid pCR-GlPDE1cat. To get a plasmid containing the sequence coding for the full length protein lacking the N-terminal transmembrane helices (Met-588 –Glu-1371) of GlPDE, a further PCR product was produced using the primer pair GlPDE1-Mfor (5'-GAGCA*GTCGA****C*ATATG**CCGTGTGTTTTGTTTCGACTGG-3') and GlPDE1-LMrev (5'-GGTCTGCATGAGAACAGTACACGGTACTGAGCATG-3') and was cloned via NdeI (bold) and BsrGI into pCR-GlPDE1cat resulting in plasmid pCR-GlPDE1M. The inserts of both plasmid constructs were verified by sequencing and subcloned into two variants of the yeast expression vector pLT1 [42] using the restriction enzymes SalI (italic)/EcoRV or NdeI (bold)/EcoRV. The EcoRV cloning site originated from the multiple cloning site of the pCRII-TOPO vector. One pLT1 variant directs the expression of the *Giardia* protein alone, whereas the other adds an N-terminal hemagglutinin (HA) tag to allow detection of the recombinant protein. Transformation of the constructs into the PDE-deficient *S*. *cerevisiae* strain PP5 (MATa leu2-3 leu2- 112 ura3-52 his3-532 his4 cam pde1::URA3 pde2::HIS3) was carried out as described previously [43].

### Yeast complementation assay

The heat-shock assay to detect complementation of the PDE-deficient phenotype of the *S*. *cerevisiae* strain PP5 was carried out exactly as described [43]. Yeast strains were streaked onto SC-leu plates (selective medium lacking leucine) and grown for 2 days at 30°C. The colonies were then replica-plated onto YPD plates prewarmed to 55°C, and were incubated for another 15 min at 55°C. Plates were then cooled to room temperature and incubation was continued at 30°C for 18–36 h.

### Yeast cell extracts

Yeast cell pellets were created exactly as described previously [44]. Briefly, yeast was grown at 30°C in SC-leu medium to end log phase and thereafter again for 3.5 h in YPD to maximize protein expression. Washed cells were frozen in liquid nitrogen and stored at -70°C. Frozen cell pellets were thawed on ice and resuspended in an equal volume of ice-cold extraction buffer (50 mM HEPES, pH 7.5, 100 mM NaCl, 1x Complete protease inhibitor cocktail without EDTA (Roche Applied Science)). Cell lysates were produced by three passages through a high pressure homogenizer (French press at 20’000 psi, 4°C). Unbroken cells and cell debris were removed by centrifugation for 20 min at 16’000 RCF at 4°C. 25% glycerol was added to the cleared supernatant. Aliquots were snap-frozen in liquid nitrogen and stored at -70°C for subsequent PDE activity assays.

### PDE assay

PDE activity was determined by a modification of the two-step procedure of Thompson and Appleman [45] as described previously [42]. In all reactions no more than 20% of the substrate was hydrolyzed. Assays were always carried out in triplicates and at 1 μM cAMP concentration. Inhibitors were dissolved and diluted in dimethyl sulfoxide (DMSO), with 1% final DMSO concentration in the reaction mixes. Control reactions with DMSO alone were always included. Data were analyzed using the GraphPad Prism software package (GraphPad, San Diego).

### *G*. *lamblia* trophozoite proliferation assay

Test compounds were dissolved in DMSO and serially diluted in *Giardia* culture medium. *G*. *lamblia* WBC6 trophozoites were grown until adherent cells reached a confluency close to 100%. Cells were then chilled on ice for 20 min and adherent parasites were detached from the culture tube. Cultures were diluted to 5x10^3^ cells/ml in growth medium (room temperature) and 100 μl aliquots were immediately added to wells of 96-well plates preloaded with the same volume of test compound dilutions. DMSO (solvent) controls were always included. Plates were incubated for 72 h at 37°C in a humid chamber with an anaerobic atmosphere. Adherent cells were then washed twice with 200 μl PBS before adding 200 μl of PBS containing 10 mg/ml resazurin and 1% glucose. After incubation for 4 h at 37°C, plates were read on a fluorescence plate reader (Wallac 1420 VICTOR^2^, PerkinElmer) with excitation and emission wavelengths of 544 nm and 590 nm, respectively. Data were analyzed with the GraphPad Prism software package using a sigmoidal dose-response model for regression. All assays were done in quadruplicate and at least three independent experiments were performed.

### Transient expression of HA-tagged GlPDE in *Giardia* trophozoites

C-terminally HA-tagged GlPDE was expressed under its endogenous promotor in *G*. *lamblia* trophozoites as follows: The entire open reading frame of GlPDE (gene ID GL50803_14058) and 43bp of the 5’-upstream region were PCR amplified from genomic DNA using the primer pair 5’-CG**TCTAGA**TTTGTGTCATAAGCAAGGTAA-3’ and 5’-CG***TTAATTAA***CTACGCGTAGTCTGGGACATCGTATGGGTACTCCTTCTTGTCCAGCTCAA-3’. These primers introduce a C-terminal HA-tag (underlined) and the two restrictions sites XbaI (bold) and PacI (bold italic) into the amplicon. The XbaI/PacI-digested PCR product was cloned into vector PAC-CHA [46] and *Giardia* WBC6 trophozoites were transfected by electroporation using 15 μg of plasmid DNA as input (device and settings: Bio-Rad Gene Pulser, 350V, 960 μF, 800 Ω). Stable transfectants were selected with the antibiotic puromycin (Sigma, cat. 7699111) at a concentration of 77 μM. Expression of the PDE is driven by its endogenous promoter in the upstream region. The transiently transfected cells, expressing HA-tagged GlPDE under its endogenous promoter, were used in all localization studies.

### Peripheral vesicle (PV) labeling and immunofluorescence analysis

Endocytic uptake of the fluorescent dye “dextran Alexa Fluor 594” (Life Technologies, cat. D22913) by PV organelles was achieved as described previously [47]. Briefly, trophozoites were grown and harvested as described above. Cells were washed twice in 1x PBS (900 x g, 10 min, 4°C) and resuspended in PBS supplemented with 5 mM cysteine, 5 mM glucose, and 0.1 mM ascorbic acid containing 4 mg/ml dextran Alexa Fluor 594. Trophozoites were transferred to 37°C for 30 minutes, protected from light. Samples were then washed twice in PBS (900 x g, 10 min, 4°C), fixed in 3% formaldehyde and processed for immunofluorescence [48]. For immunofluorescence staining, a fluorescein isothiocyanate (FITC)-conjugated mouse anti-HA antibody (dilution 1:100; Roche Diagnostics GmbH, Manheim, Germany) was used. Prior to inspection, specimens were embedded in Vectashield (Vector Labs, Inc, cat. H-1200) containing the DNA intercalating agent 49-6-Diamidino-2-phenylindole (DAPI). Immunofluorescence analysis was performed on a Nikon Eclipse 80i microscope using the software Openlab 5.5.2 (PerkinElmer) for picture acquisition.

### RNA preparation and reverse transcription

RNA was isolated from wild type cells using an RNAeasy kit (QIAGEN Cat. 74104) following the ‘Animal Cells Spin’ protocol. A DNase I digestion was included to remove residual genomic DNA according to the manufacturer’s protocol. RNA was eluted in 50 μl of RNase-free water. Reverse transcription was performed using a Qiagen Omniscript RT kit (Cat. 205111) and cDNA was amplified by PCR using different combination of primers.

## Results

### The *G*. *lamblia* genome encodes only a single class I PDE

The vast majority of genes in the *Giardia* Genome Database (www.GiardiaDB.org version 4.0) [49] were annotated based on automated annotation algorithms. However, *Giardia* is a highly diverged organism [50]. To enhance the detection power for a putative PDE, we performed a comprehensive search in GiardiaDB using the HMMER software package (v 3.1b). To identify class I PDEs, a profile hidden Markov model (HMM) was built from the catalytic domain of 24 PDE sequences, including members of all eleven hPDE families as well as a selection from phylogenetically diverse species. This search identified a single class I PDE (GlPDE; e-value = 4e-90) in all *G*. *lamblia* isolates sequenced so far (gene IDs GL50803_14058, DHA2_150867, GL50581_303, GSB_153349, GLP15_4333). A putative PDE with similarity to GlPDE was also identified in the database of annotated proteins of the salmon parasite *Spironucleus salmonicida*, a diplomonad species such as *G*. *lamblia*. GlPDE and the *Spironucleus* PDE (SsPDE, GiardiaDB gene ID SS50377_13952) share a similar domain structure, a short stretch of weak homology in the N-terminal protein half (185 aa with 24% identity) and 35% identity in the C-terminal catalytic domain, indicating that both enzymes are distantly related members of the same PDE family (see S2 Fig). To identify class II or class III PDEs [12] in *G*. *lamblia*, the database was queried with the appropriate profile HMMs (see Materials and methods), but no corresponding protein sequences could be detected. To validate the automated annotation of the GlPDE open reading frame, RNA from trophozoites in exponential growth phase was reverse transcribed, and the presence of the full length GlPDE transcript was confirmed. No substantial alterations in the RNA expression levels during *in vitro*–induced trophozoite-to-cyst differentiation were found in a recently published RNA-seq study [51]. In contrast, the putative nucleotidyl cyclases identified in the GiardiaDB by means of profile HMM searches (S1A Fig), as well as the protein kinase A subunits (gPKAr and gPKAc) display a differential expression pattern during in vitro encystation (S1B Fig).

#### Domain organization of GlPDE

**Fig 1:**
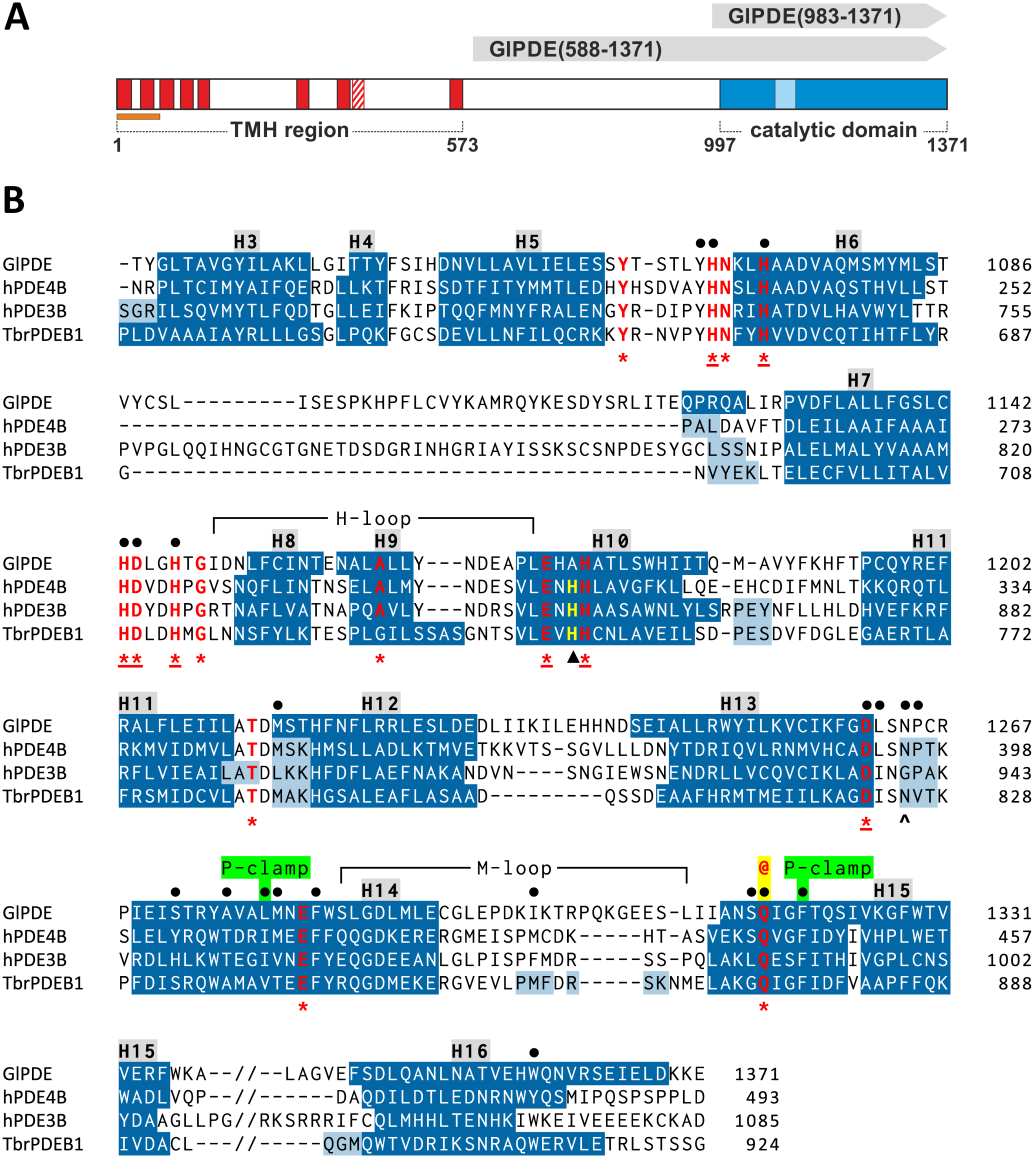
Domain structure of GlPDE and alignment of its catalytic domain sequence with other PDEs. **(A)** Schematic representation of the domain structure. The predicted transmembrane helices (TMHs) between amino acids 1 and 573 are shown as red boxes. One segment is predicted with less confidence (predicted by 4 of 14 algorithms) and is depicted as a hatched box. The C-terminal catalytic domain is shown in blue and the GlPDE-specific 35aa-insert between helix 6 and 7 is indicated by a light-blue box. The N-terminal 71 amino acids that were recognized by the program MitoProtII as mitochondrial-targeting signal are indicated with an orange bar. GlPDE regions 588–1371 and 983–1371 corresponding to the recombinant proteins expressed in yeast are marked with grey arrows. **(B)** Sequence alignment of the catalytic domains (α-helices 3–16) of GlPDE, human PDE4B, human PDE3B and *T*. *brucei* TbrPDEB1. Secondary structure features are shown in dark blue (α-helices) and light blue (3_10_-helices). α-helices are additionally numbered above the sequences (H3-H16). Residues of the substrate-binding subpocket are indicated with dots above the alignment. Thereof, the invariant glutamine is additionally marked with an @-sign (highlighted in yellow) and the hydrophobic P-clamp residues are tagged with green labels. Amino acids that are conserved in all 11 human PDE families and in GlPDE are shown in red letters and indicated with an asterisk below the alignment. Asterisks are underlined at the eight strictly conserved residues of the metal-binding pocket at the site of catalysis. The single histidine residue that is conserved among all hPDEs, but is substituted by an alanine in GlPDE (Ala-1175) is marked with a black triangle.

The open reading frame of GlPDE (GL50803_14058) encodes a protein of 1371 amino acids (molecular mass = 154.9 kDa, pI = 7.4; see Fig 1A). The N-terminal region (aa 1–573) is predicted to contain eight or nine transmembrane helices (TMH). Interestingly, the first 71 residues, comprising two TMHs, are reminiscent for a predicted mitochondrial targeting signal (MitoProt II [52], p = 0.96). No significant similarity to any sequence in the TrEMBL database could be found within the 420 amino acids between the last TMH and the catalytic domain (aa 574–996). The highly conserved catalytic domain is located at the C-terminus (aa 997–1371; see Fig 1A). In accordance with average homology between *Giardia* genome assemblages A, B and E [50], GlPDE homologs show an overall sequence identity of 77 to 90% (S1 File). Lowest sequence conservation is found in the 140 aa -long region between TMH 5 and 6 (35 to 73% identity) and the highest one in the catalytic domain (86 to 94%). All residues of the substrate-binding pocket in the catalytic domain are fully conserved between the three *Giardia* genotypes, with the only exception of Ser-1316 that is replaced by a leucine in isolate P15 of the non-human infective assemblage E (S1 File).

#### Catalytic domain

The catalytic domain of GlPDE is predicted to conform closely to those of other class I PDEs of human or parasite origin. It is predicted to form 16 alpha helices that align well with those of human and kinetoplastid PDEs (Fig 1B). Sequence identity to the human PDEs varies from 21% (hPDE6A) to 32% (hPDE4D), which is in the range usually found between different PDE families (S1 Table). All functionally important residues are highly conserved between GlPDE and the consensus sequence of human PDEs (see S3 Fig). This includes the purine-scanning invariant glutamine (Gln-1317) and a pair of residues forming a hydrophobic clamp around the nucleobase (P-clamp [53,54] Leu-1279 and Phe-1329), as well as the eight strictly conserved residues of the metal-binding pocket at the site of catalysis (His-1068, His-1072, His-1143, Asp-1144, His-1147, Glu-1173, His-1176, Asp-1261).

However, GlPDE also contains some notable differences to the human enzymes. First, a 35 amino acid sequence is inserted into the loop between helix 6 and 7, reminiscent of an insert also found in hPDE3 isozymes, but the two sequences are not related. Secondly, the GlPDE catalytic domain contains a unique M-loop connecting helices 14 and 15 (Figs 1B and S5). The M-loop contributes to the catalytic pocket in human PDEs and is also involved in shaping the P-pocket of kinetoplastid PDEs and the related “selectivity pocket” of hPDE10, which both are exploited for inhibitor design [55–57]. The M-loop of GlPDE is the least conserved part of the catalytic domain as determined by conservation scoring functions (trident/valdar01 [31]).

Interestingly, the H-loop contains a residue, Asp-1151, which is unique in GlPDE in terms of its physico-chemical properties since all hPDEs contain a polar but uncharged amino acid (Asn,Thr or Ser) at this position (S3 Fig). In various human and kinetoplastid PDEs the corresponding residue forms a part of the catalytic pocket and has been recognized as an anchor point for inhibitors to increase isozyme selectivity [58–63].

A further prominent difference between GlPDE and the human PDEs is found outside of the catalytic cleft (residues Gly-1146 and Ala-1175). These two residues substitute a totally conserved acidic residue (usually aspartic acid) and an invariant histidine of human PDEs, respectively (S3 Fig), which have been implicated in forming a structure-stabilizing salt bridge [64] between the ends of the H-loop. Most importantly, the M-loop and the H-loop are completely conserved between the sequenced *Giardia* assemblages A, B and E (S1 File).

### GlPDE functionally complements a PDE-deficient *S*. *cerevisiae* strain and hydrolyzes cAMP, but not cGMP *in vitro*

**Fig 2:**
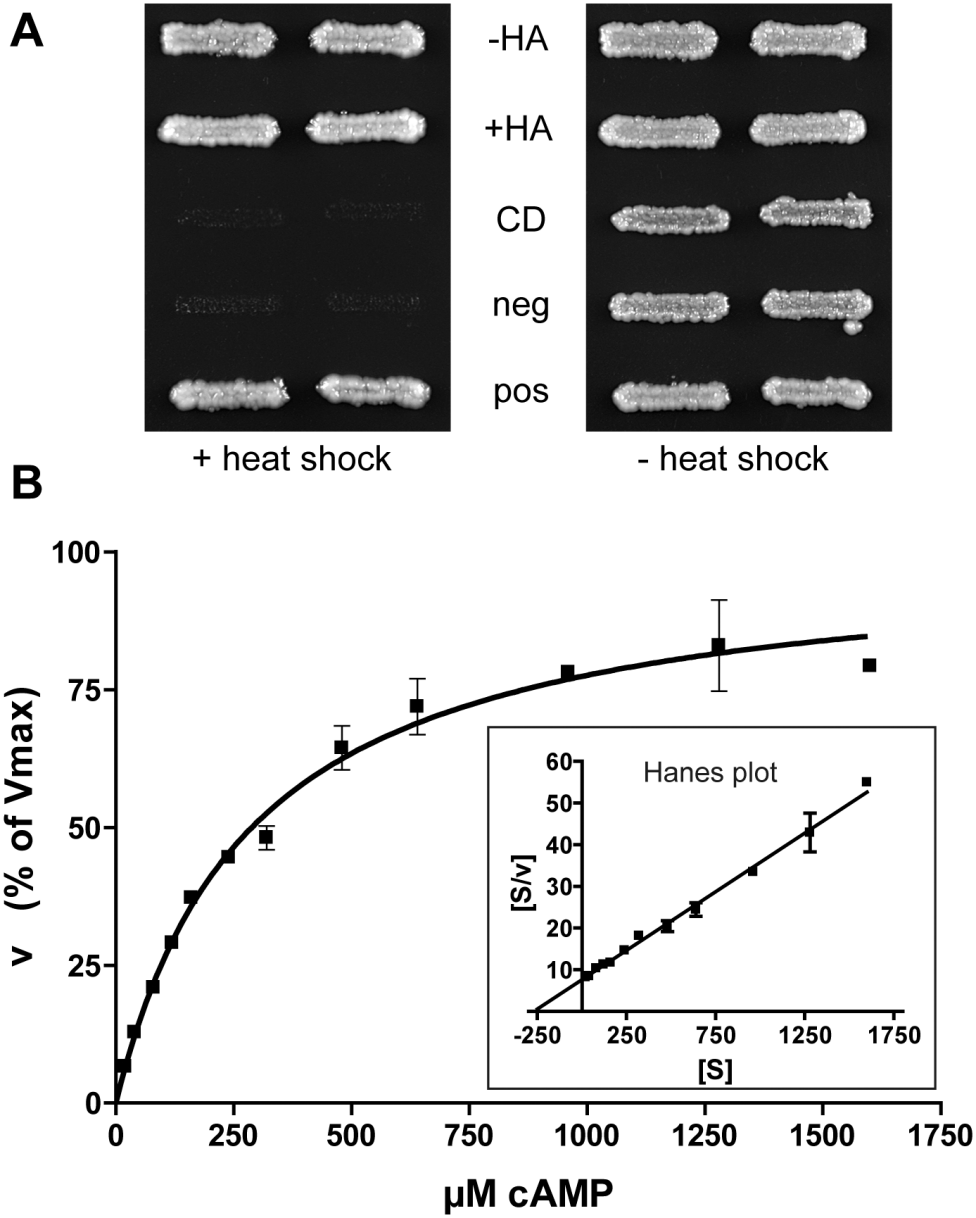
PDE activity of recombinant GlPDE. **(A)** The heat-shock phenotype of the PDE-deletion yeast strain PP5 is complemented by GlPDE. Duplicate patches of recombinant yeast strains were subjected to an initial heat shock at 55°C for 15 min (left, +heat shock) or not (right, -heat shock), and were then grown at 30°C for 2 days.–HA: PP5 expressing GlPDE(aa588-1371); +HA: PP5 expressing GlPDE(aa588-1371) containing an N-terminal hemagglutinin (HA) tag; CD: PP5 expressing GlPDE(aa983-1371); neg: PP5 transfected with the empty expression vector (negative control); pos: PP5 expressing the catalytic domain of human PDE4D (positive control). **(B)** Michaelis-Menten kinetics of recombinant GlPDE(aa588-1371) with cAMP as substrate. Insert: Corresponding Hanes plot. All data point determinations were done in triplicate. Square symbols represent the mean values, and error bars the standard error of the mean (SEM). The shown graph is a representative example of four experiments.

*S*. *cerevisiae* strains lacking both endogenous PDEs display heat-shock sensitivity as a phenotype [65]. Functional complementation of this phenotype by heterologous PDE expression has proven to be a sensitive tool to validate PDE enzyme function [66,43,42]. Since transmembrane domains tend to interfere with recombinant expression of proteins, a GlPDE construct was used that contained the full catalytic domain, but lacked the N-terminal transmembrane domains (amino acids Met-588 –Glu-1371) (see Fig 1A). The construct was expressed in the PDE-deficient yeast strain PP5 [66]. As predicted from sequence analysis, the recombinant protein was catalytically active, and it fully restored heat-shock resistance of the mutant yeast strain, also in the presence of an N-terminal HA tag (see Fig 2A). Expression of a truncated version (see Fig 1A) comprising the catalytic domain solely (Gly-983 to Glu-1371) did not confer heat-shock resistance to the mutant strain.

Soluble lysates prepared from the recombinant yeasts expressing GlPDE(aa588-1371) hydrolyzed cAMP with standard Michaelis-Menten kinetics with a K_m_ of 408 ± 37 μM (Fig 2B). The enzyme is cAMP-specific and does not accept cGMP as a substrate. No cAMP-hydrolyzing activity could be detected in lysates from the parental PP5 strain.

### Effect of PDE inhibitors on GlPDE activity and on *Giardia* trophozoite proliferation

**Fig 3:**
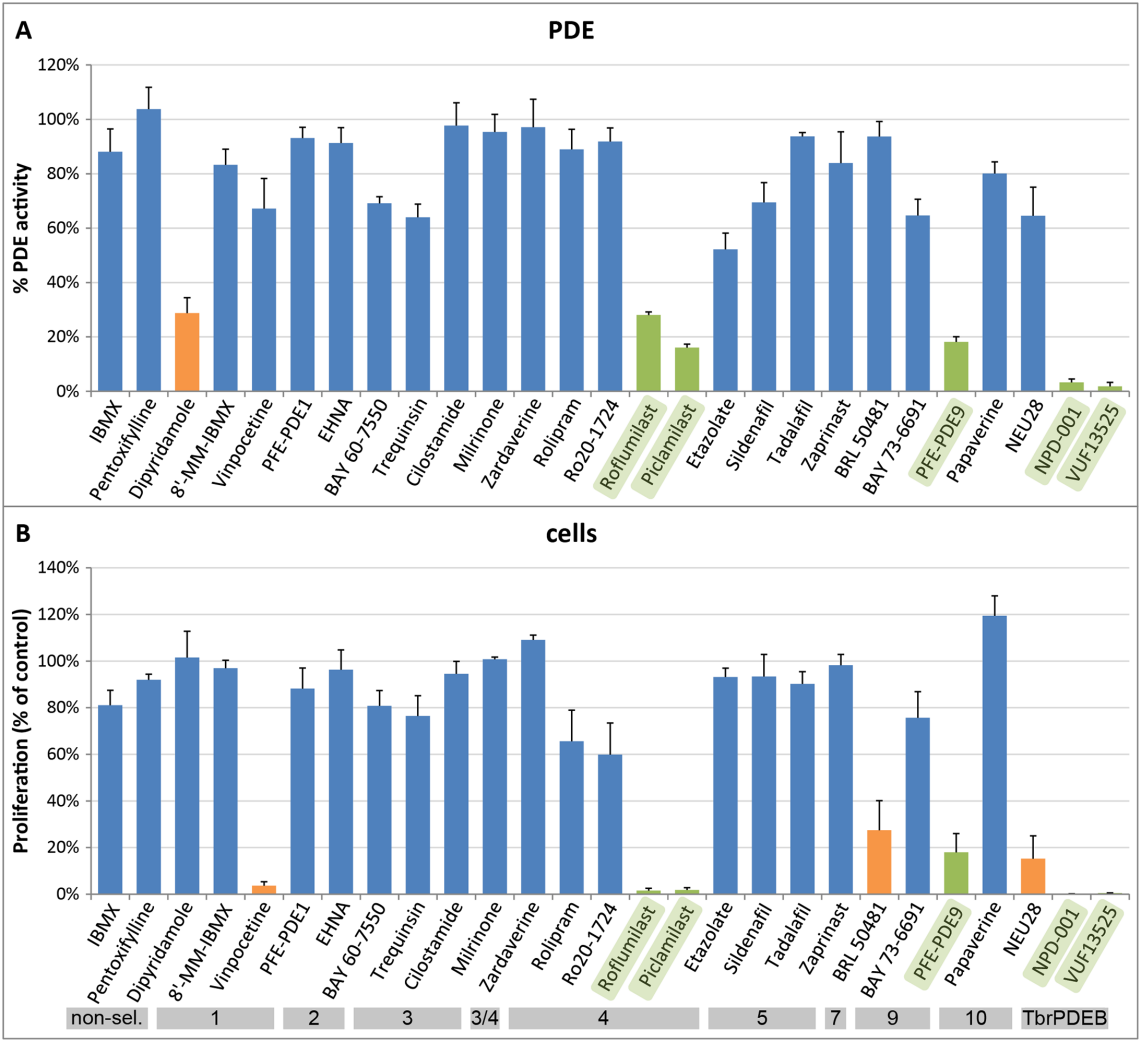
Effect of human and trypanosomal PDE inhibitors on GlPDE activity and *Giardia* trophozoite proliferation. **(A)** Inhibitors were tested at 100 μM against recombinant GlPDE(aa588-1371) enzyme. **(B)** Trophozoite proliferation after 72h culture in the presence of 50 μM inhibitor. Compounds that are effective against trophozoites and also impair enzyme activity are indicated with green bars, those inhibiting only either throphozoite proliferation or GlPDE activity are presented with orange bars. Selectivity of the compounds for different PDE families is shown below the graphs using grey horizontal bars (non-sel. = non-selective; 1–10 = hPDE1-10; TbrPDEB = *T*.*brucei* TbrPDEB1/B2).

**Fig 4:**
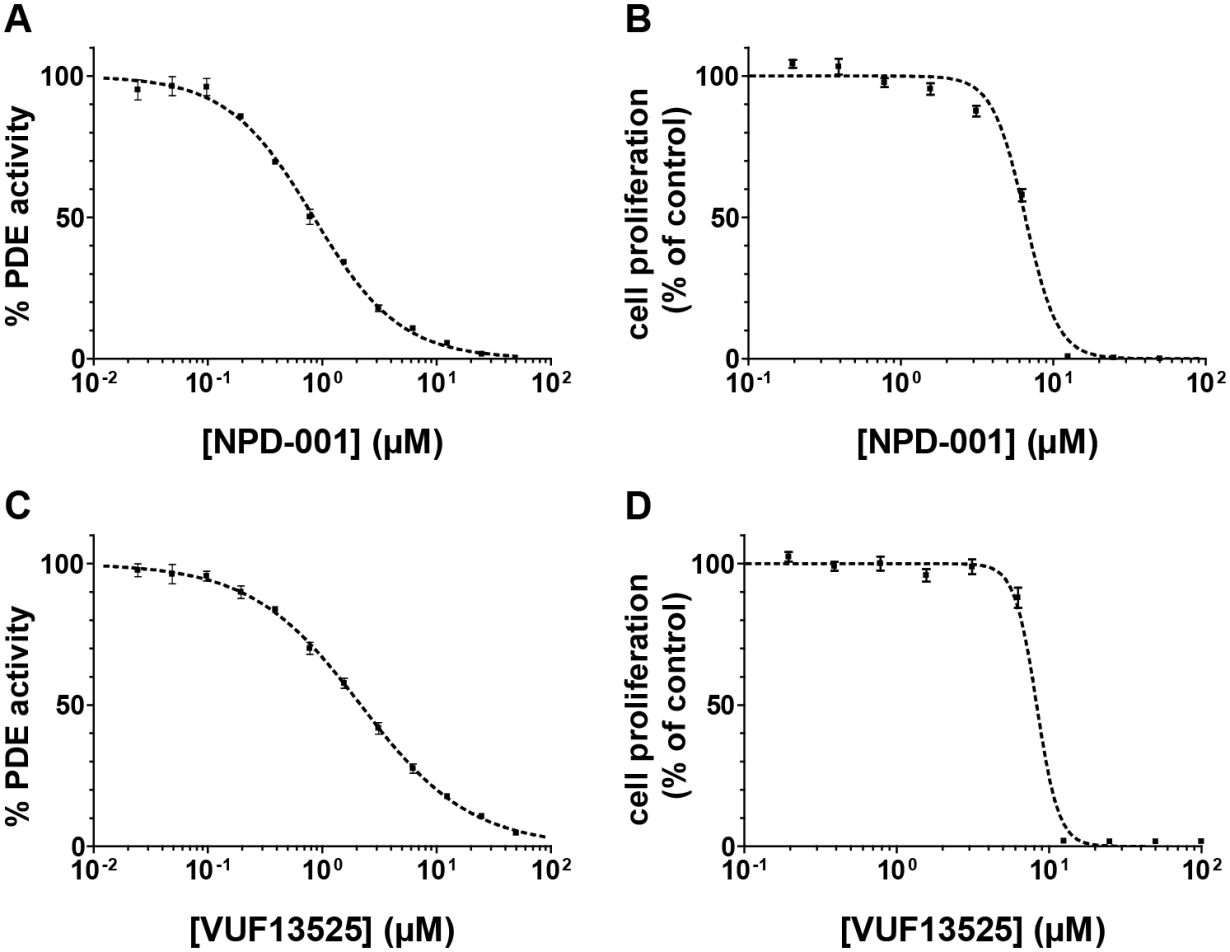
Representative dose-response curves of the most active compounds. Panels **(A)** and **(C)** show IC_50_ determinations for NPD-001 and VUF13525 against recombinant GlPDE(aa588-1371) enzyme (substrate concentration: 1 μM); Panels **(B)** and **(D)** show the dose-dependent effect of the same compounds on proliferation of *G*. *lamblia* trophozoites (WB, clone 6), determined after 72 h of anaerobic in vitro culture. Square symbols represent the mean values of determinations done in triplicate **(A,C)** or quadruplicate **(B,D)**. Error bars represent the standard error of the means (SEM).

To pharmacologically validate GlPDE, a selection of 27 PDE inhibitors covering specific human and trypanosomal PDE families were assessed with cell lysates of recombinant yeast strain PP5 expressing GlPDE(aa588-1371). This profiling assay showed that most of the hPDE-family-specific inhibitors were essentially inactive against recombinant GlPDE, even at 100 μM concentration (Fig 3A). In contrast, the two trypanosomal PDE inhibitors NPD-001 and VUF13525, as well as the compounds dipyridamole, PFE-PDE9 (PubChem SID 15099396), roflumilast, piclamilast, and NEU222 showed promising activity against GlPDE. The same inhibitors were assessed for their activity against the proliferation of *G*. *lamblia* trophozoites in culture (Fig 3B). At 50 μM final concentration, most drugs showed no or only minor anti-giardial activity. However, those compounds that were active against recombinant GlPDE (roflumilast, piclamilast, PFE-PDE9, NPD-001 and VUF13525) also inhibited *Giardia* trophozoite proliferation, with the exception of the non-selective inhibitor dipyridamole. These data indicate a strong correlation between anti-giardial activity and GlPDE enzyme inhibition. To confirm our findings, the IC_50_ values of the compounds found to be active as shown in Fig 3, and additionally the piclamilast analog NEU222, were determined against recombinant GlPDE (aa588-1371) and compared to the EC_50_ values obtained in the cell proliferation assays. Again, there is a good agreement between their potency for enzyme inhibition and for anti-parasitic activity (Table 1). Representative dose-response curves of the most active compounds NPD-001 and VUF13525 are given in Fig 4.

**Table 1:** Potency of selected PDE inhibitors. From those compounds exhibiting IC_50_ <100 μM against recombinant GlPDE (aa588-1371) and EC_50_ <50 μM against *Giardia* trophozoites in the benchmark screening (see in Fig 3) exact dose responses were determined (values highlighted in green). PDE activity assays were done in triplicates and repeated at least once (SE of logIC_50_ < = 0.1), and *Giardia* cell susceptibility assays were done in quadruplicates and repeated 2 to 5 times (SE of logEC_50_ < = 0.15). n.d., not determined.

inhibitor	human PDE selectivity	Giardia PDE IC_50_ (μM)	Giardia cells EC_50_ (μM)
IBMX	n.s.	>100	>50
Pentoxifylline	n.s.	>100	>50
Dipyridamole	5,6,10,11	26.3	>50
8'-MM-IBMX	1	>100	>50
Vinpocetine	1	>100	16.3
PFE-PDE1	1	>100	>50
EHNA	2	>100	>50
BAY 60–7550	2	>100	~50
Trequinsin	3	>100	>50
Cilostamide	3	>100	>50
Milrinone	3	>100	>50
Zardaverine	3,4	>100	>50
Rolipram	4	>100	>50
Ro20-1724	4	>100	>50
Roflumilast	4	6.0	7.4
Piclamilast	4	11.3	12.0
NEU222	4	5.1^#^	5.9
Etazolate	4	~100	>50
Sildenafil	5	>100	>50
Tadalafil	5	>100	>50
Zaprinast	5	>100	>50
BRL 50481	7	>100	n.d.
BAY 73–6691	9	>100	>50
PFE-PDE9	9	5.8	15.1
Papaverine	10	>100	>50
NEU28	10	~100	30.6
NPD-001	4, TbrPDEB	0.8	6.9
VUF13525	TbrPDEB	2.3	9.4

^#^Bottom of the dose-response curve constrained to the background level.

### The localization pattern of transiently overexpressed GlPDE is reminiscent of mitosomes and the perinuclear region

**Fig 5:**
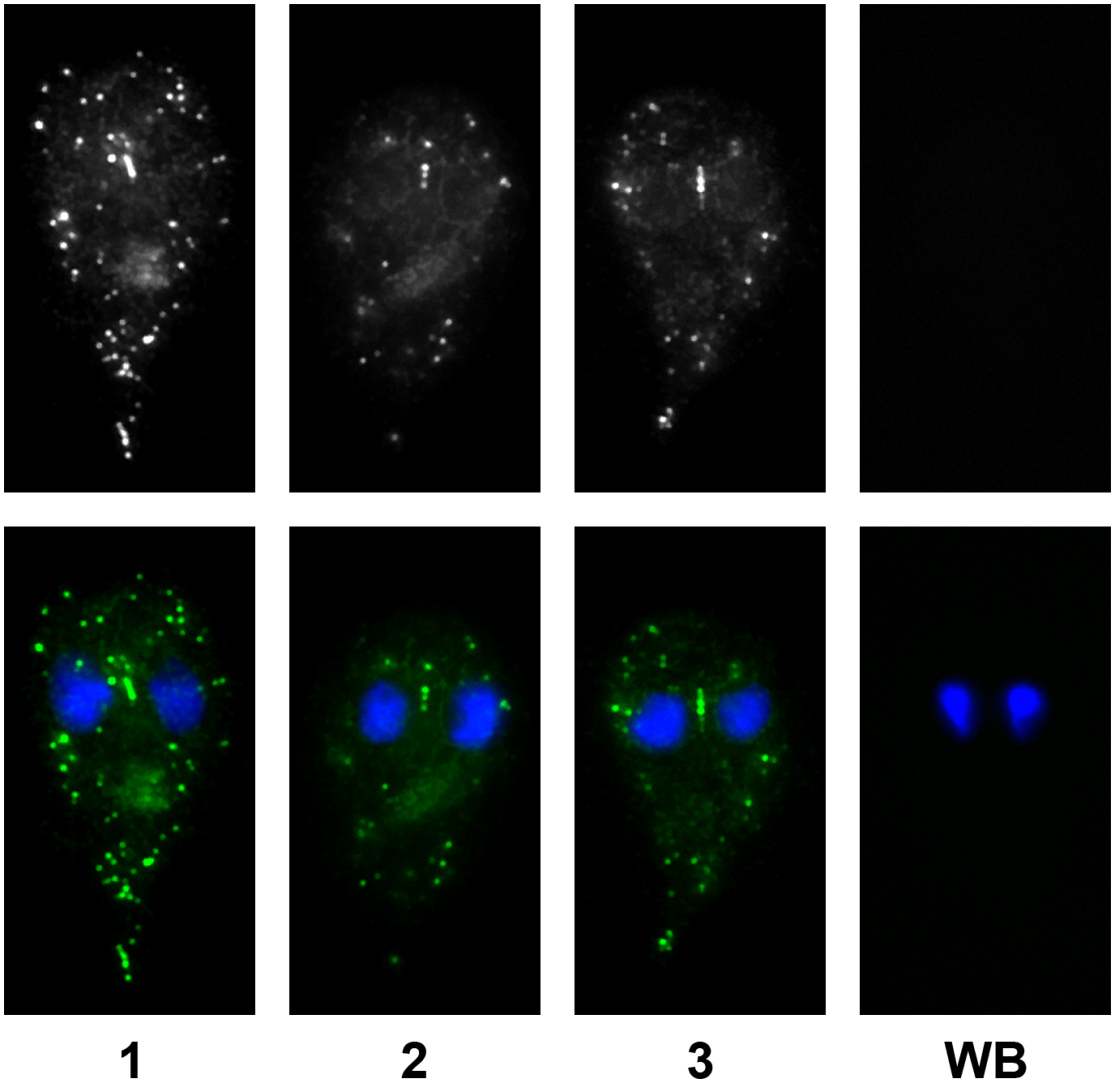
Localization of GlPDE in *Giardia* trophozoites. C-terminally HA-tagged GlPDE was expressed under its endogenous promotor in *Giardia lamblia* trophozoites and detected with a FITC-conjugated anti-HA antibody. Cells of two independent transient transfections were analyzed by microscopy. An average of 50.6% of the cells were positively stained with the FITC-conjugated antibody and showed signal in discrete dots in the periphery of the cells and in the perinuclear region. In around 40% of the cells, the chimeric protein also aligned as—generally three—discrete dots between the two DAPI-stained nuclei, reminiscent of the central mitosomes. Upper row: Three representative transfected cells (1–3) and wildtype control cell (WB, clone C6). Lower row: Overlay of the same GlPDE-HA staining (green) with DAPI-stained nuclei (blue).

In order to investigate the localization of GlPDE in *G*.*lamblia* trophozoites, parasites were transfected with a plasmid encoding a C-terminally HA-tagged copy of GlPDE plus the 200 bp upstream region containing the putative endogenous promotor, resulting in the transiently transfected strain PDEp-PDEHA. The subcellular localization of the chimeric protein was detected by immunofluorescence staining in formaldehyde-fixed cells using an anti-HA antibody. The HA-tagged GlPDE was predominantly detected in dot-like patterns in the trophozoite periphery, and at distinct dots that are centrally located between the two nuclei. This localization pattern may indicate that GlPDE is associated with the various peripheral and the centrally located mitosomes. Further staining was found in the region surrounding the *Giardia* nuclei, which is reminiscent for the endoplasmic reticulum (see Fig 5) [67].

**Fig 6:**
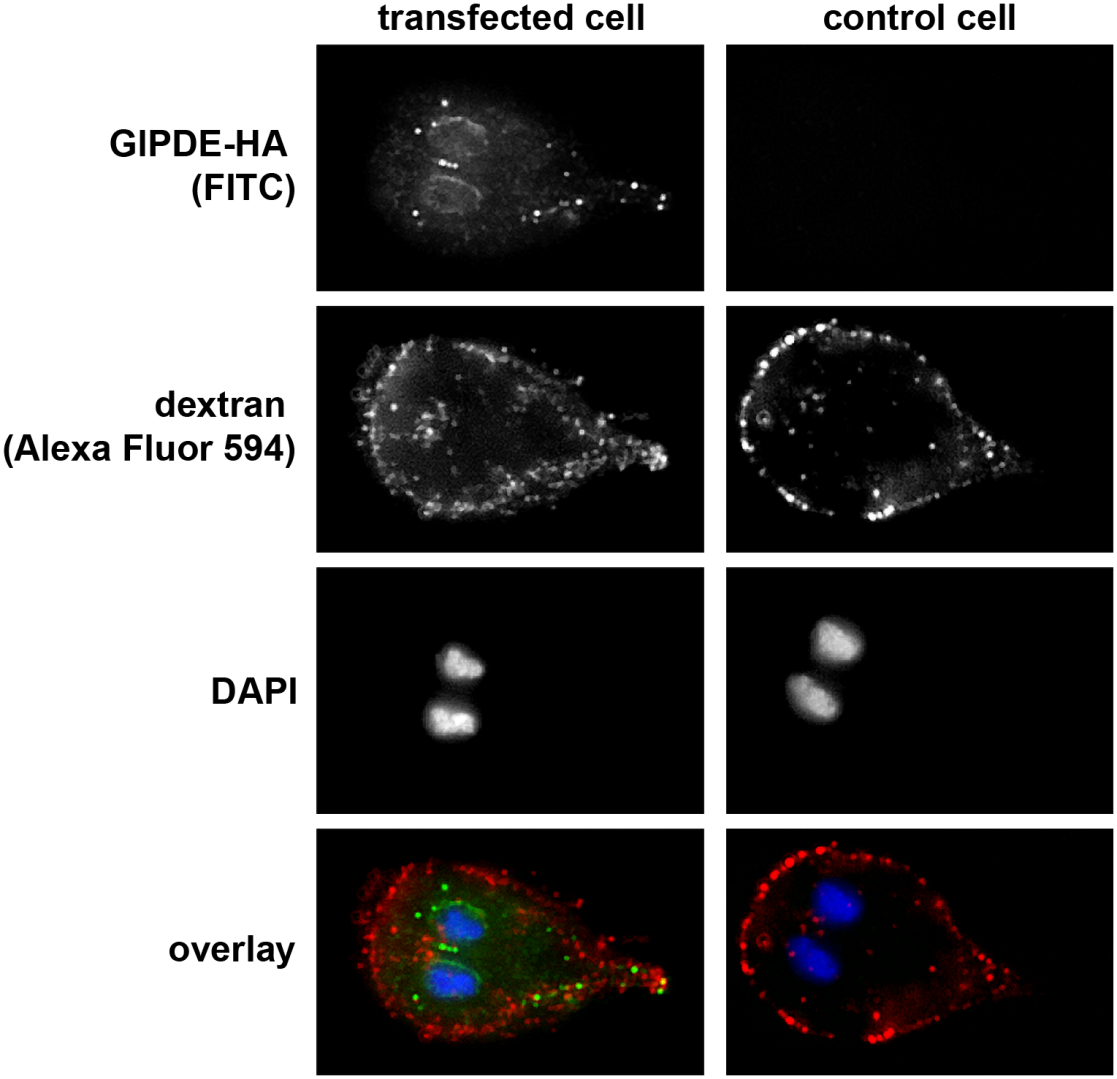
Differential localization pattern of GlPDE-HA and internalized dextran in peripheral vesicles. Peripheral vesicles were loaded with Alexa Fluor 594-coupled dextran (red) and GlPDE-HA was detected with a FITC-conjugated anti-HA antibody (green). Nuclei were stained with DAPI (blue). The overlay of images shows completely different patterns of green and red signals. Left column represents a transfected cell, right column shows a non-transfected control cell.

To exclude that GlPDE was localized in endocytic peripheral vesicles (PVs), the transiently transfected *Giardia* strain PDEp-PDEHA was first incubated in the presence of Dextran coupled to Alexa Fluor 594 (red), followed by fixation and staining with anti-HA antibodies and a secondary antibody conjugated to Alexa Fluor 488 (green) (see Fig 6). Microscopy clearly showed that the red PV staining and the green HA staining signals did not overlap. PVs were located more distally and were located closely underneath the plasma membrane, whereas the green anti-HA-labelled structures were found throughout the cell, but were less abundant in the cellular periphery.

## Discussion

The present study reports on the identification and characterization of the single PDE from *G*. *lamblia*, GlPDE, and it addresses the question whether this enzyme could represent a valid drug target. The sequence characteristics unambiguously classify GlPDE as a new member of the class I PDE superfamily that includes all human and many protozoan parasite PDEs [68,27]. GlPDE exhibits many strongly conserved features with the human PDEs, but is also clearly distinct from them.

The N-terminal half of the protein is predicted to contain eight or nine putative transmembrane helices (TMH), of which the first two are predicted to represent a mitochondrial targeting signal. The TMH region is followed by a stretch of about 400 amino acids with no discernible domains, and a C-terminal catalytic domain with a high degree of similarity to those of the human PDEs.

The presence of such an extended N-terminal transmembrane domain containing two long extra-membranous regions (~140aa) is a feature that has not been described in other PDEs to date, though membrane-anchoring through a TMH bundle is also found in many other PDEs such as in hPDE3 [69] and in all five *Plasmodium falciparum* PDEs [70].

The prediction of an N-terminal mitochondrial targeting signal in GlPDE is in agreement with our finding, that HA-tagged GlPDE transiently expressed in *Giardia* trophozoites suggests a localization at mitosomes. Mitosomes are organelles that are found in unicellular microaerophilic or anaerobic organisms such as *Entamoeba*, microsporidia and *Giardia*. They are believed to be derived from mitochondria, but lack the capability to gain energy from oxidative phosphorylation [71,72]. A number of mitosome-associated proteins are closely related to those of mitochondria [73]. Our tentative association of GlPDE with mitosomes may represent a further similarity with mitochondria of higher eukaryotes, where cAMP synthesis and hydrolysis is an important regulatory circuit for mitochondrial metabolism [74,75]. Since mitosomes do not occur in mammalian cells, but are essential for *Giardia* and presumably regulated by cAMP signaling, future studies to verify the localization of GlPDE are certainly warranted.

With respect to the catalytic domain, all residues important for substrate recognition and catalysis are conserved between GlPDE and the consensus of human PDEs (S3 Fig). Substrate specificity of PDEs is determined by a set of approximately twelve residues that define shape and chemical nature of the subpocket that accommodates the nucleobase [76,77,15]. Concerning these residues, GlPDE conforms best to the cAMP-specific PDEs (S4 Fig). This is in full agreement with our experimental findings that recombinant GlPDE is cAMP-specific and does not accept cGMP as a substrate. The observed K_m_ for cAMP of 408 μM is much higher when compared with hPDEs, where values do not exceed 20 μM for the preferred cyclic nucleotide [68]. Although we cannot exclude an artifact of recombinant expression in yeast, class I PDEs with a high K_m_ for their preferred substrate have been identified in other lower eukaryotes: For the PDEA orthologs of various kinetoplastid parasites, K_m_ values between 190 and 600 μM have been reported [78,43,79].

The high K_m_ of recombinant GlPDE(aa588-1371) for cAMP indicates that cAMP hydrolysis in *Giardia* occurs with first-order kinetics over a wide concentration range. The presence of a high-K_m_ PDE as the sole enzyme for cyclic nucleotide degradation is somewhat unusual. However, a similar case is observed for the fission yeast *Schizosaccharomyces pombe*. Its genome codes only for a single class II PDE, Cgs2, that exhibits a high K_m_ for cAMP. Nevertheless, this single PDE apparently is sufficient for all aspects of cAMP management, a situation akin to *G*. *lamblia* [80,81].

Other unicellular eukaryotes that express high-K_m_ PDEs usually also express a low-K_m_ enzyme, though their distinct roles are often not clearly understood. In the yeast *S*. *cerevisiae*, the high-K_m_, class II PDE, pde1, is the major regulator of agonist-induced cAMP signaling, while the low-K_m_, class I PDE, pde2, has only a limited effect on this pathway [82]. In the slime mold *Dictyostelium discoideum*, several discrete pools of cAMP exist in the cell, and each is addressed by a specific pair of PDEs, one of high K_m_ and low capacity, and the second of low K_m_ and high capacity. In combination, they are thought to keep the average half-life of cAMP down, irrespective of fluctuating local concentrations [83]. In the fungal rice pathogen, *Magnaporthe oryzae*, the high-K_m_ PdeH and the low-K_m_ PdeL play unique and non-redundant roles, with PdeH involved in conidial morphogenesis and PdeL in pathogenic development [84,85].

In order to pharmacologically evaluate the role of GlPDE, a panel of established PDE inhibitors with activity against human and/or trypanosomal PDEs was screened against recombinant GlPDE (aa 588–1371; lacking the N-terminal TMHs). We identified a number of compounds that inhibited GlPDE in the low micromolar or sub-micromolar concentration range. All these inhibitors, with the exception of dipyridamole, also impaired *Giardia* trophozoite proliferation. Interestingly, the compounds with good correlations of biochemical and cell-biological activities represent four different chemical scaffolds, and thus represent an excellent starting base for their further development into *Giardia*-specific PDE inhibitors and potential drug candidates. In addition, the amino acid sequence differences, and—by inference—the corresponding structural differences, between human and *Giardia* PDEs, indicate that a discrimination between human PDEs and GIPDE by appropriate structure optimization of drug candidates is feasible.

Not surprisingly, a few compounds of the original set exhibited an effect on trophozoite proliferation, but did not inhibit GlPDE enzyme activity, indicating off-target effects. It could also mean that such compound targets only the native full-length PDE in the parasite and not the heterologously expressed GlPDE(aa588-1371) used in the screenings. However, this is rather unlikely, since almost all known PDE inhibitors target the PDE catalytic pocket [15]. In the case of BRL50481, the inhibitory effect on trophozoite proliferation may be attributed to the compound’s nitro-group. Anaerobic organisms such as *Giardia* reduce nitro-groups to toxic nitroso derivatives, as it is the case for metronidazole, the current first-line drug against giardiasis [86]. Off-target effects are also well known for established inhibitors of human PDEs. Vinpocetine, a classical hPDE1 inhibitor, also co-targets several proteins in addition to PDE1, such as voltage sensitive Na+ -channels and IKK, a “mediator” of NF-κB action [87–89]. Thus, this compound is no longer considered a specific PDE inhibitor in whole-cell assay formats [90].

In conclusion, we show that *G*. *lamblia* expresses only a single PDE, which we here characterized on the molecular and functional level. A first pharmacological validation has shown that targeting GlPDE activity leads to significant impairment of *G*. *lamblia* throphozoite proliferation *in vitro*, which would be an important prerequisite to alleviate giardial disease symptoms. These promising findings warrant further studies *in vitro* and *in vivo* to exploit GlPDE as a potential target for the treatment of giardiasis.

### Accession numbers

The GiardiaDB (http://www.giardiadb.org) accession numbers for the gene products discussed in this paper are as follows: (i) GlPDE homologs in different *Giardia* isolates (assemblage/isolate): A/WB (GL50803_14058), A/DH (DHA2_150867), B/GS (GL50581_303), B/GS_B (GSB_153349), E/P15 (GLP15_4333); (ii) identified putative nucleotidyl cyclases (classIII) in assemblage A, isolate WB: strong hit (GL50803_14367), strong hit (GL50803_16599), weak hit (GL50803_16492); (ii) protein kinase A subunits (assemblage A, isolate WB): catalytic subunit gPKAc (GL50803_11214), regulatory subunit gPKAr (GL50803_9117); (iii) putative phosphodiesterase SsPDE of *S*. *salmonicida* strain ATCC50377 (SS50377_13952).

## Supporting information

S1 Fig Transcriptional profiles of putative nucleotidyl cyclases (NCs), GlPDE and gPKA subunits during *in vitro*—Encystation. (PDF)

S2 Fig Comparison of the domain structure and amino acid sequence of *Giardia lamblia* GlPDE and *Spironucleus salmonicida* SsPDE. (PDF)

S3 Fig Alignment of catalytic pocket and conserved residues. (PDF)

S4 Fig Residues that have been recognized to be important for substrate specificity (cAMP versus cGMP). (PDF)

S5 Fig Alignment of the M-loop region of different PDEs. (PDF)

S1 Table Sequence identity in the catalytic core between GlPDE and other PDEs. (PDF)

S1 File Alignment of the GlPDE homologs from all five sequenced *G*.*lamblia* isolates. (PDF)
