# Supplementary material for: The single cyclic nucleotide-specific phosphodiesterase of the intestinal parasite Giardia lamblia represents a potential drug target
Source: PLoS Negl Trop Dis. 2017 Sep 15;11(9):e0005891. doi: 10.1371/journal.pntd.0005891 (PMC5617230; doi:10.1371/journal.pntd.0005891)
Supplement: S1 Fig — (PDF) [file pntd.0005891.s001.pdf]

S1 Fig. Transcriptional profiles of putative nucleotidyl cyclases (NCs), GlPDE and gPKA subunits during *in vitro* – encystation.

A

GiardiaDB ID	Description	e-value	bit score
GL50803_14367	putative NC	2.2E-36	124.0
GL50803_16599	putative NC	2.8E-27	94.4
GL50803_16492	putative NC (weak hit)	5.7E-03	15.3

B

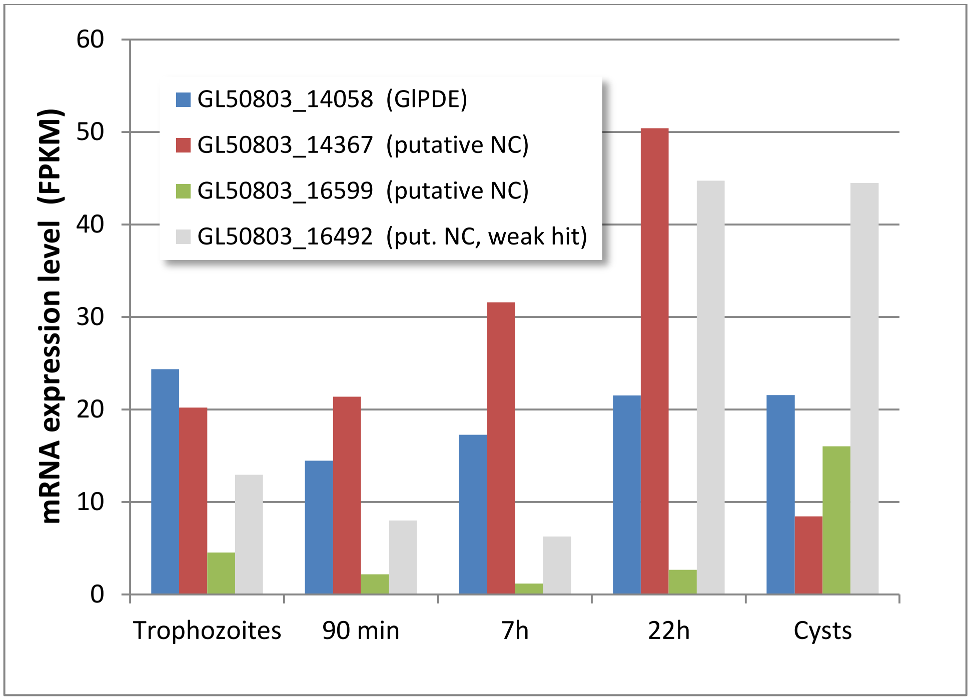

C

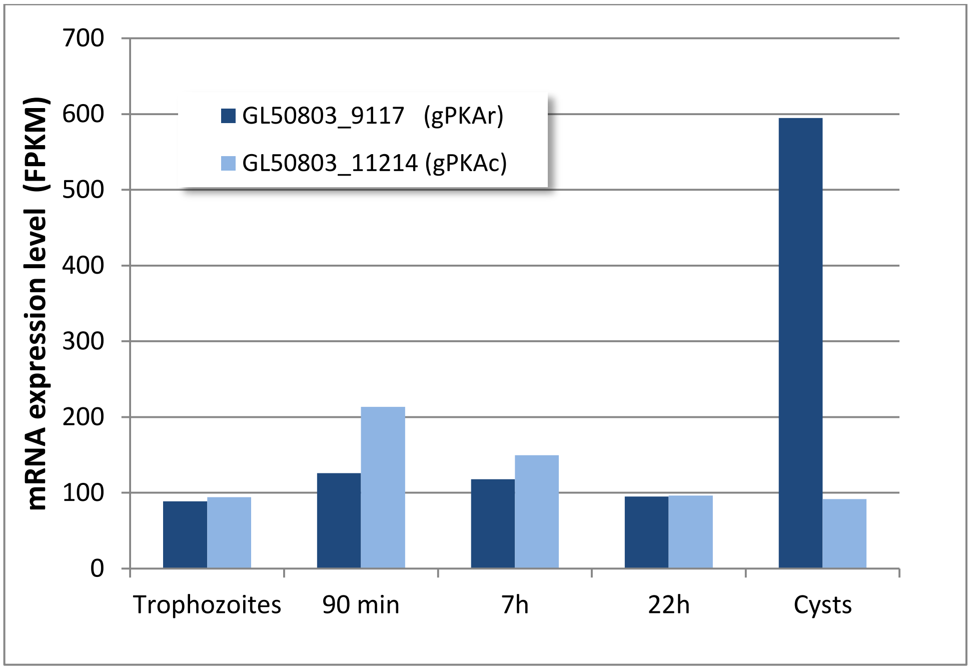

LEGEND

(A) Identification of putative *G. lamblia* class III nucleotidyl cyclases (NCs). Using profile hidden Markov models and the program hmmsearch of the HMMER software suite, three putative NCs were identified in the GiardiaDB (v31) (e-value inclusion threshold = 0.01). The GiardiaDB sequences GL50803_14367 and GL50803_16599 represent strong hits, whereas GL50803_16492 is only a weak hit. The table shows the e-values and bit scores of the three hits. **(B)** mRNA expression levels of GlPDE (GL50803_14058) and the putative NCs during *in vitro* -induced encystation. **(C)** mRNA expression levels of the regulatory subunit gPKAr (GL50803_9117) and the catalytic subunit gPKAc (GL50803_11214) of protein kinase A during in vitro induced encystation.

All shown FPKM values (Fragments Per Kilobase of transcript per Million mapped reads) were extracted from the RNA-seq data set of Einarsson et al (2016) [1]. While expression of GlPDE does not change more than twofold during encystation, the mRNA concentration of all putative NCs and the gPKA subunits alters significantly (fold change > 2) during differentiation to cysts.

Reference:

[1] Einarsson E, Troell K, Hoeppner MP, Grabherr M, Ribacke U, Svärd SG. Coordinated Changes in Gene Expression Throughout Encystation of Giardia intestinalis. PLoS Negl Trop Dis. 2016; 10:e0004571. doi:10.1371/journal.pntd.0004571. pmid: 27015092
