# Supplementary material for: The single cyclic nucleotide-specific phosphodiesterase of the intestinal parasite Giardia lamblia represents a potential drug target
Source: PLoS Negl Trop Dis. 2017 Sep 15;11(9):e0005891. doi: 10.1371/journal.pntd.0005891 (PMC5617230; doi:10.1371/journal.pntd.0005891)
Supplement: S2 Fig — (PDF) [file pntd.0005891.s002.pdf]

S2 Fig. Comparison of the domain structure and amino acid sequence of *Giardia lamblia* GlPDE and *Spironucleus salmonicida* SsPDE

(A) Domain structures

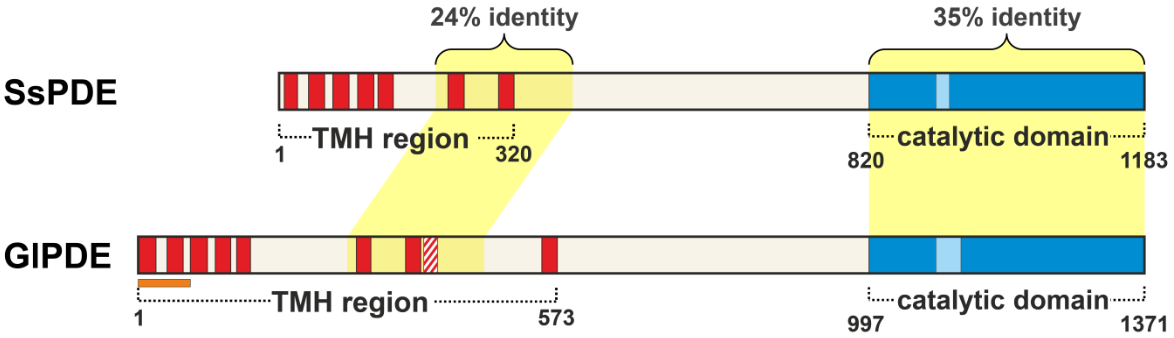

LEGEND

The predicted transmembrane helices (TMHs) in the N-terminal protein regions are shown as red boxes. One segment in GlPDE is predicted with less confidence (by 4 of 14 algorithms) is depicted with a hatched box. The 71 amino acids at the N-terminus of GlPDE that were recognized by the program MitoProtII as mitochondrial-targeting signal (p=0.96) are indicated with an orange bar. The program did not identify such a targeting signal in the sequence of SsPDE (p=0.24). The C-terminal catalytic domains are shown in blue. The GlPDE-specific 35aa-insert and the SsPDE-specific 18aa-insert between helix 6 and 7 are indicated by a light-blue box. The inserts in GlPDE and SsPDE do not show any sequence similarity (see sequence alignment). Regions in GlPDE and SsPDE that were found to show sequence similarity are highlighted in yellow: (i) GlPDE aa 280-464 and SsPDE aa 215-399 show 24% identity, (ii) the catalytic domains share 35% sequence identity.

(B) Sequence alignments

Similarity region in the N-terminal protein part:

```
                                         putative TMH
GlPDE/280-464      HRFAEAPPINFFPHIEPTMFYLVTVSLLFVTALGLNVCYKYLLRGKLMEIL---PQLIHF       336
SsPDE/215-399      QRLYRQLPKNIFPKFQGLPLIIFIFNVI---TLA-LIIYDFYLQIVLIRIFENSSNLIKI       270
                   :*: .  * *:**:::   : :. ..::   :*.  : *.: *:  *:.*:    :**::

                                                      putative TMH
GlPDE/280-464      THKSTESE--YMSSSASIYATFLEFINNYNIFPAMHAILILQMIFIVPLCNGNTVDVMMV       394
SsPDE/215-399      YQSATNDKVKWAYALVEAQKSLVLFDKLYMYVPILFAFVCSS-VLAIFVSLSSKPKIENV       329
                    :.:*:.:  :  : ..   ::: * : *  .* :.*::  . :: : :. ... .:  *

                    weak TMH hit
GlPDE/280-464      FTNPVIFQVFARWLESNRFGAPLAFLIDCYFYRQRPSYERMIVNKIYVKWIDMLANGMLP       454
SsPDE/215-399      FENEVLFKLFAGWLYFNRCQNLLLFLQDSFLLKTQFTIDTTTANIIFIKYQDLVQSGELS       389
                   * * *:*::** **  **    * ** *.:: : : : :   .* *::*: *:: .* *

GlPDE/280-464      CTGIALSEVE  464
SsPDE/215-399      ISRHAFNQLD  399
                    :  *:.:::
```

Catalytic domain (helices 3 – 16) :

```
                        +----H3----+   +H4    +----H5-----+      ●● +-●--H6----
GlPDE/1021-1371    YTRTYGLTAVGYILAKLLGITTYFSIHDNVLLAVLIELESSYTSTLYHNKLHAADVAQMS       1080
SsPDE/848-1183     LSKGHCALAVTFLAIKYLDIDVLLQIPDTALFACLYELEQGYTPTLYHNRLHIADFVQMV       907
                    :: :   ** ::  * *.* . :.* *..*:* * ***..** *****:** **..**

                   ----+ |~~~~~~~~~~~~~insert~~~~~~~~~~~~~~|        +---H7-----
GlPDE/1021-1371    MYMLSTVYCSLISESPKHPFLCVYKAMRQYKESDYSRLITEQPRQALIRPVDFLALLFGS       1140
SsPDE/848-1183     FLQILAINAQYSAFV-----------------KQTSFEQSASQREIILSPTDIFSLIMAA       950
                   :  : :: ..  :                   .: *   : . *: :: *.*:::*::.:
```

```
                            |<======H-Loop=======>|
                   -+●●  ●     +-H8-+ +-H9-+     +-----H10-----+          +-H11
GlPDE/1021-1371    LCHDLGHTGIDNLFCINTENALALLYNDEAPLEHAHATLSWHIITQMAVYFKHFTPCQYR       1200
SsPDE/848-1183     CCHDFGHTGIDNPFCINSQNVAAIIYNDIGPMEQAHASLSWGLVSKFSTIFLNWSVPQFR       1010
                    ***:******* ****::*. *::*** .*:*:***:*** ::::::. * :::  *:*

                   ---H11----+   ●------H12-----+            +-------H13-------
GlPDE/1021-1371    EFRALFLEIILATDMSTHFNFLRRLESLDEDLIIKILEHHND----SEIALLRWYILKVC       1256
SsPDE/848-1183     EFRIKFIELVLATDMTFHFPFVQKITKISSNYLMEYFDRFKHNKINQRDQIFKWFVMKCI       1070
                   ***  *:*::*****: ** *:::: .:..: ::: :::.:.    ..  :::*:::*

                                      %      |<=========M-Loop========>|
                   ----●● ●●   +--●---●--●●--●--H14---+        ●              +-●
GlPDE/1021-1371    IKFGDLSNPCRPIEISTRYAVALMNEFWSLGDLMLECGLEPDKIKTRPQKGEESLIIANS       1316
SsPDE/848-1183     MKFGDLANPTRSFPVAEYWAYSYINEQRITGDIMRELNWPPNLILNPNKR--DLDFLCGS       1128
                   :*****:** * : ::  :* : :**    **:* * .  *: * .  ::  :  ::..*

GlPDE/1021-1371    QIGFTQSIVKGFWTVVERFWKA------LAGVEFSDLQANLNATVEHWQNVRSEIELDKK       350
SsPDE/848-1183     QIGFVNFVLRPFLGEINRLVFKASTLTKLDYCLYAKLQQNMVNTSEIWEERNAKK-----       336
                   ****.: ::: *   ::*:         *    ::.** *:  * * *:: .::

                   @  %
                   ●--●-----H15------+            +-------H16---●---------+
GlPDE/1021-1371    QIGFTQSIVKGFWTVVERFWK------ALAGVEFSDLQANLNATVEHWQNVRSEIELDKKE      1371
SsPDE/848-1183     QIGFVNFVLRPFLGEINRLVFKASTLTKLDYCLYAKLQQNMVNTSEIWEERNAKK            1183
                   ****.: ::: *   ::*:         *    ::.** *:  * * *:: .::
```

LEGEND

Matches in the alignments (shown below the sequences):

* = all residues in that column are identical

: = conserved substitutions

. = semi-conserved substitutions

Sequence annotations:

<----Hx----> = alpha helix number x in the catalytic domain

<==========> = loops involved in forming or lining the catalytic pocket (H-loop, M-loop)

|~~~~~~~~~~| = insert in GlPDE (35aa) and SsPDE (18aa) between helix 6 and 7 of the catalytic domain

● = residue of the substrate-binding pocket

@ = invariant glutamine

% = P-clamp residue

Y = P-clamp residue of SsPDE, deviating from all class I PDEs that have been characterized so far.

Q = glutamine residue of SsPDE that is replacing a conserved phenylalanine/tyrosine in the substrate-binding pocket of hPDEs.

A = an alanine residue in GlPDE and SsPDE that is replacing a strictly conserved histidine in hPDEs. The histidine has been implicated in forming a salt bridge to a conserved acidic amino acid in front of the H-loop. This acidic residue of hPDEs is itself substituted by a glycine (G) in both diplomonad PDEs.
