# Supplementary material for: The single cyclic nucleotide-specific phosphodiesterase of the intestinal parasite Giardia lamblia represents a potential drug target
Source: PLoS Negl Trop Dis. 2017 Sep 15;11(9):e0005891. doi: 10.1371/journal.pntd.0005891 (PMC5617230; doi:10.1371/journal.pntd.0005891)
Supplement: S3 Fig — (PDF) [file pntd.0005891.s003.pdf]

# S3 Fig. Alignment of catalytic pocket and conserved residues

| | | | | | | | | | | | | | | | | | | | | | |
|---|---|---|---|---|---|---|---|---|---|---|---|---|---|---|---|---|---|---|---|---|---|
| | | | | | z | z | zm | | | | | | | | | | | | | | z |
| | | | | | | | | | | in H-loop | | | | | | | | | | | |
| Pocket (Card2004): | | Q | M | | M | M | M | M | S | S | M | | M | M | | | | | M | M | M |
| active site (Ke2011): | | x | x | | x | x | x | x | | | | | | | | | | | | x | x |
| GlPDE1 residue: | 1062 | 1067 | 1068 | 1069 | 1072 | 1143 | 1144 | 1147 | 1149 | 1151 | 1152 | 1163 | 1172 | 1173 | 1175 | 1176 | 1212 | 1213 | 1214 | 1215 | 1261 |
| **GlPDE** | Y | Y | H | N | H | H | D | H | G | **[D]** | N | A | L | E | **[A]** | H | A | T | D | M | D |
| hPDE1 | Y | Y | H | N | H | H | D | H | G | T | N | A | L | E | H | H | A/S | T | D | M | D |
| hPDE2 | Y | Y | H | N | H | H | D | H | G | N | N | A | M | E | H | H | A | T | D | L | D |
| hPDE3 | Y | Y | H | N | H | H | D | H | G | T | N | A | L | E | H | H | A | T | D | L | D |
| hPDE4 | Y | Y | H | N | H | H | D | H | G | S | N | A | L | E | H | H | A | T | D | M | D |
| hPDE5 | Y | Y | H | N | H | H | D | H | G | N | N | A | M | E | H | H | A | T | D | L | D |
| hPDE6 | Y | Y | H | N | H | H | D | H | G | N | N | A | L | E | H | H | A | T | D | L | D |
| hPDE7 | Y | Y | H | N | H | H | D | H | G | N | Q | A | L | E | H | H | A | T | D | I | D |
| hPDE8 | Y | Y | H | N | H | H | D | H | G | T | N | A | L | E | H | H | A | T | E | M | D |
| hPDE9 | Y | F | H | N | H | H | D | H | G | N | N | A | L | E | H | H | A | T | D | M | D |
| hPDE10 | Y | Y | H | N | H | H | D | H | G | S | N | A | M | E | H | H | A | T | D | L | D |
| hPDE11 | Y | Y | H | N | H | H | D | H | G | N | N | A | L | E | H | H | A | T | D | L | D |
| TbrPDEB1 | Y | Y | H | N | H | H | D | H | G | N | N | G | L | E | H | H | A | T | D | M | D |
| LmjPDEB1 | Y | Y | H | N | H | H | D | H | G | N | N | G | L | E | H | H | A | T | D | M | D |
| TcrPDEC1 | Y | Y | H | N | H | H | D | H | G | T | N | S | L | E | L | H | H | T | D | M | D |
| PfPDE B | Y | Y | H | T | H | H | D | H | G | N | N | A | L | E | Y | H | S | T | D | M | D |
| PfPDE | Y | Y | H | N | H | H | D | H | G | N | N | S | L | E | Y | H | S | T | D | M | D |
| PfPDE | Y | Y | H | N | H | H | D | H | G | T | N | S | L | E | Y | H | A | T | D | M | D |
| PfPDE | Y | Y | H | N | H | H | D | H | G | T | N | S | L | E | Y | H | S | T | D | M | D |
| consensus hPDEs: | Y | YF | H | N | H | H | D | H | G | NTS | NQ | A | LM | E | H | H | AS | T | DE | LMI | D |

| | | | | | | | | | | | | | | | | | | | | | | | |
|---|---|---|---|---|---|---|---|---|---|---|---|---|---|---|---|---|---|---|---|---|---|---|---|
| | | | | | | | P-clamp1 | | | | | | | | | | | | invariant Q | P-clamp2 | | | |
| | | | | | | | | | | | in M-loop | | | | | | | | | | | | |
| Pocket (Card2004): | Q | Q | Q | Q | Q | Q | Q | Q | S | S | S | | | S | Q | S | Q | Q | Q | Q | | | |
| active site (Ke2011): | x | x | s | s | | s | s | s | | s | | | | | s | | | s | s | s | s | x | s |
| GlPDE1 residue: | 1262 | 1264 | 1265 | 1272 | 1275 | 1276 | 1279 | 1280 | 1282 | 1283 | 1286 | 1288 | 1290 | 1298 | 1300 | 1301 | 1313 | 1316 | 1317 | 1320 | 1321 | 1324 | 1358 |
| **GlPDE** | L | N | P | S | **[Y]** | A | L | M | E | F | **[L]** | D | **[M]** | **[D]** | I | **[K]** | I | S | Q | F | **[T]** | I | W |
| hPDE1 | I | H | P | H | W | T | L | M/L | E | F | Q | D | E | S | L | C | V | S | Q | F | I | I | W |
| hPDE2 | L | D | Q | T | I | A | I | Y | E | F | Q | D | E | M | M | M | I | L | Q | F | M | I | W |
| hPDE3 | I | G | P | H | W | T | I | V | E | F | Q | D | E | S | F | M | L | L | Q | F | I | I | W |
| hPDE4 | L | N | P | Y | W | T | I | M | E | F | Q | D | E | S | M | C | V | S | Q | F | I | I | Y |
| hPDE5 | L | A | I | Q | I | A | V | A | E | F | Q | D | E | T | L | M | I | M | Q | F | I | I | W |
| hPDE6 | L | A | I | Q | V | A | V | A | E | F | Q | D | E | I | M | M | L | L | Q | F | I | V | W |
| hPDE7 | I | N | P | S | W | S | V | T/C | E | F | Q | D/E | E | S | L | C | I | I | Q | F | M | L/I | W |
| hPDE8 | V | N | P | C | W | A | I | S | E | Y | Q | D | E | V | V | F | I | S | Q | F | I | F | W |
| hPDE9 | I | N | E | A | W | V | L | L | E | Y | Q | D | E | A | F | M | K | A | Q | F | I | V | Y |
| hPDE10 | L | S | V | T | T | A | I | Y | E | F | E | D | M | I | M | M | V | G | Q | F | Y | V | W |
| hPDE11 | L | A | V | S | V | A | V | T | E | F | Q | D | E | S | I | F | L | L | Q | W | I | I | W |
| TbrPDEB1 | I | N | V | S | W | A | V | T | E | F | Q | D | E | L | M | F | L | G | Q | F | I | V | W |
| LmjPDEB1 | V | N | V | S | W | A | V | T | E | F | Q | D | E | L | M | F | L | G | Q | F | I | V | W |
| TcrPDEC1 | I | A | S | A | W | L | I | L | E | F | Q | E | E | T | G | F | V | S | Q | F | L | F | Y |
| PfPDE B | I | H | S | H | W | T | I | N | E | F | Q | L | E | S | L | C | L | S | Q | F | L | L | W |
| PfPDE | I | H | G | H | W | C | V | L | E | F | Q | D | E | S | L | C | V | S | Q | F | L | V | W |
| PfPDE | L | H | N | H | W | V | L | V | E | F | E | D | L | N | L | F | F | I | Q | F | L | L | W |
| PfPDE | I | H | T | H | W | T | V | S | E | F | Q | R | E | D | L | N | I | T | Q | F | F | I | W |
| consensus hPDEs: | LIV | NAG HSD | | | WIVT | | IVL | | E | FY | QE | DE | EM | | MLF VI | MCF | ILV K | | Q | FW | IMY | IVF L | WY |

Amino acid residues are colored according to the Clustal color scheme. Assignments to sub-regions of the catalytic pocket as defined by Card et al (2004) and Ke et al (2011) are given above the alignment, **M**: metal-binding pocket, **Q**: hydrophobic pocket, **S**: solvent-filled pocket, **x,s**: active-site residues whereof s denotes the substrate-specificity pocket. The four metal-binding residues are marked by m (Mg-coordinating) and z (Zn-coordinating). The invariant glutamine and the residues forming the P-clamp are indicated accordingly, as are amino acids belonging to the H- and the M-loop. The human consensus sequence is shown below the alignment, and GlPDE residues deviating from the human consensus are displayed in black boxes. All listed amino acids are conserved among the three sequenced *Giardia* assemblages A, B and E, with the exception of S1316, which is a leucine in assemblage E.

**Color scheme:**

| amino acids | property |
|---|---|
| AVFPMILW | Small (small+ hydrophobic (incl.aromatic -Y)) |
| DE | Acidic |
| RK | Basic - H |
| STYHCNGQ | Hydroxyl + sulfhydryl + amine + G |

**References**


Card GL, England BP, Suzuki Y, Fong D, Powell B, Lee B, et al. Structural basis for the activity of drugs that inhibit phosphodiesterases. Structure. 2004;12: 2233–47. doi:10.1016/j.str.2004.10.004

Ke H, Wang H, Ye M. Structural insight into the substrate specificity of phosphodiesterases. Handb Exp Pharmacol. 2011; 121–34. doi:10.1007/978-3-642-17969-3_4
