# Supplementary material for: The single cyclic nucleotide-specific phosphodiesterase of the intestinal parasite Giardia lamblia represents a potential drug target
Source: PLoS Negl Trop Dis. 2017 Sep 15;11(9):e0005891. doi: 10.1371/journal.pntd.0005891 (PMC5617230; doi:10.1371/journal.pntd.0005891)
Supplement: S4 Fig — (PDF) [file pntd.0005891.s004.pdf]

S4 Fig. Residues that have been recognized to be important for substrate specificity (cAMP versus cGMP)

Aligned are the residues of kinetically characterized PDEs (1 PDE per family). Species abbreviations in PDE names are: h, human; Dm, *Drosophila melanogaster* ; Tbr, *Trypanosoma brucei* ; Tcr, *Trypanosoma cruzi* ; Lmj, *Leishmania major* ; Ldo, *Leishmania donovani* ; Pf, *Plasmodium falciparum* ; Sc, *Saccharomyces cerevisiae* ; Dd, *Dictyostelium discoideum* .

		asparagine region			substrate-specificity pocket according to Ke et al (2011)											
		asparagine o |			o	o	o	P-clamp1 |	o				invariant Q |	P-clamp2 |	o	o
	GlPDE residue:	1264	1067	1075	1265	1272	1276	1279	1280	1283	1300	1316	1317	1320	1321	1358
	GlPDE	**N**	Y	D	P	S	A	L	M	F	I	S	**Q**	F	T	W
**cAMP -specific**	hPDE4	**N**	Y	D	P	Y	T	I	M	F	M	S	**Q**	F	I	Y
	hPDE7	**N**	Y	D	P	S	S	V	T/C	F	L	I	**Q**	F	M	W
	hPDE8	**N**	Y	D	P	C	A	I	S	Y	V	S	**Q**	F	I	W
	DmPDE4 (dunce)	**N**	F	D	P	Y	V	L	M	F	M	S	**Q**	F	I	Y
	TbrPDEA	**N**	Y	D	C	Y	S	V	S	F	F	G	**Q**	F	I	G?
	TbrPDEB1	**N**	Y	D	V	S	A	V	T	F	M	G	**Q**	F	I	W
	LmjPDEB1	**N**	Y	D	V	S	A	V	T	F	M	G	**Q**	F	I	W
	LdoPDED	**A**	Y	D	Q	A	L	V	V	M	G	G	**Q**	F	M	?
	ScPDE1	**N**	F	D	V	S	A	I	T	F	V	G	**Q**	F	I	W
	DdPDE2	**N**	Y	D	I	N	S	V	S	F	F	I	**A**	F	I	W
	DdPDE4	**N**	Y	D	P	Y	S	V	T	F	F	C	**Q**	F	I	?
**dual substrate**	hPDE1	**H**	Y	D	P	H	T	L	M/L	F	L	S	**Q**	F	I	W
	hPDE2	**D**	Y	S	Q	T	A	I	Y*	F	M	L	**Q**	F	M	W
	hPDE3	**G**	Y	D	P	H	T	I	V	F	F	L	**Q**	F	I	W
	hPDE10	**S**	Y	T	V	T	A	I	Y	F	M	G	**Q**	F	Y	W
	hPDE11	**A**	Y	N	V	S	A	V	T	F	I	L	**Q**	W	I	W
	TcrPDEC	**A**	Y	D	S	A	L	I	L	F	G	S	**Q**	F	L	Y
	LmjPDEC	**A**	Y	D	Q	A	L	I	V	F	G	G	**Q**	F	M	Y
**cGMP -specific**	hPDE5	**A**	Y	N	I	Q	A	V	A	F	L	M	**Q**	F	I	W
	hPDE6	**A**	Y	N	I	Q	A	V	A	F	M	L	**Q**	F	I	W
	hPDE9	**N**	Y	C	E	A	V	L	L	Y	F	A	**Q**	F	I	Y
	PfPDE B	**H**	Y	Q	S	H	T	I	N	F	L	S	**Q**	F	L	W
	DdPDE3	**N**	F	A	E	S	A	L	M	F	F	T	**Q**	F	I	W

= structure(s) solved

o = positions that constrain the conformation of the invariant Q in different PDEs:

= H-bonding to the invariant Q

= water-mediated H-bonding to the invariant Q

= participating in H-bond network that fixes the invariant Q conformation

* = H-bond only found with bound GMP, but not with AMP

N = asparagine that can form bidentate H-bonds with AMP

YD = Y+D implied in stabilizing the asparagine side chain conformation

P = proline that positively affects the ability of N to **N** to form H-bonds with cAMP

= exclusively present in GlPDE

Substrate specificity of PDEs is determined by a set of residues that define the shape and chemical nature of the subpocket accommodating the nucleobase [1]. Among these residues two positions in conjunction with their scaffolding residues have been recognized to play a pivotal role [2,3]: First, the invariant glutamine (e.g. Q443 in hPDE4B2B) and second, a position that is occupied by an asparagine in almost all cAMP-specific PDEs (N395 in hPDE4B2B). This asparagine is thought to form bidentate hydrogen bonds to adenine and Zhang et al showed that the optimal adenine-binding conformation is stabilized by an H-bond network in hPDE4 (residues Y233 and D241 in hPDE4B2B). This 3-residue-configuration is only found in in cAMP-specific PDEs so far (see "asparagine region"). Moreover, a proline neighboring the asparagine in the polypeptide chain may further support the cAMP-binding conformation [2]. All four amino acids are present in GlPDE (N1264, Y1067, D1075, P1265). Furthermore, the side chain of the invariant glutamine of GlPDE (Q1317) is probably not constrained to an hPDE5/9-like conformation that would favor cGMP-binding (see positions, which constrain the conformation of the invariant glutamine in different PDEs, and which are marked with a circle above the alignment). Thus, these sequence characteristics indicate a preference for cAMP over cGMP in GlPDE.

References: **1.** Ke H, Wang H, Ye M. Structural insight into the substrate specificity of phosphodiesterases. Handb Exp Pharmacol. 2011; 121–34. doi:10.1007/978-3-642-17969-3_4 **2.** Zhang KY, Card GL, Suzuki Y, Artis DR, Fong D, Gillette S, et al. A glutamine switch mechanism for nucleotide selectivity by phosphodiesterases. Mol Cell. 2004;15: 279–86. doi:10.1016/j.molcel.2004.07.005 **3.** Lau JK, Cheng Y-KK. An update view on the substrate recognition mechanism of phosphodiesterases: a computational study of PDE10 and PDE4 bound with cyclic nucleotides. Biopolymers. 2012;97: 910–22. doi:10.1002/bip.22104
