# Supplementary material for: The single cyclic nucleotide-specific phosphodiesterase of the intestinal parasite Giardia lamblia represents a potential drug target
Source: PLoS Negl Trop Dis. 2017 Sep 15;11(9):e0005891. doi: 10.1371/journal.pntd.0005891 (PMC5617230; doi:10.1371/journal.pntd.0005891)
Supplement: S5 Fig — (PDF) [file pntd.0005891.s005.pdf]

S5 Fig. Alignment of the M-loop region of different PDEs. Amino acids are colored according to their chemical properties as described in figure S3. The M-loop is indicated above the sequences, so is the invariant glutamine and residues forming the hydrophobic P-clamp. The single residue of the M-loop that locates in the substrate-specificity pocket at the active site in all structurally characterized PDEs is labeled with an asterisk. Helices 14 and 15 of the catalytic domain are also indicated above the sequences using brown boxes. Amino acids of GlPDE that strongly deviate from the hPDE consensus are shown in black boxes.

			P-clamp				P-clamp1b													M-Loop																								invariant Q			P-clamp
			|				|	1285			helix 14														*												1311			helix 15			|			|
GlPDE	V	A	L	M	N	E	F	W	S	L	G	D	L	M	L	-	E	C	G	L	E	P	D	-	K	I	K	T	R	P	Q	K	G	E	E	S	-	L	I	I	A	N	S	Q	I	G	F
PDE1B1	K	A	L	M	E	E	F	F	R	Q	G	D	K	E	A	-	E	L	G	L	P	F	S	-	P	L	C	D	R	-	-	-	-	-	T	S	-	T	L	V	A	Q	S	Q	I	G	F
PDE2A3	E	L	I	Y	K	E	F	F	S	Q	G	D	L	E	K	-	A	M	G	N	R	P	M	-	E	M	M	D	R	-	-	-	-	-	E	K	-	A	Y	I	P	E	L	Q	I	S	F
PDE3B	E	G	I	V	N	E	F	Y	E	Q	G	D	E	E	A	-	N	L	G	L	P	I	S	-	P	F	M	D	R	-	-	-	-	-	S	S	-	P	Q	L	A	K	L	Q	E	S	F
PDE4D2	D	R	I	M	E	E	F	F	R	Q	G	D	R	E	R	-	E	R	G	M	E	I	S	-	P	M	C	D	K	-	-	-	-	-	H	N	-	A	S	V	E	K	S	Q	V	G	F
PDE5A1	E	L	V	A	T	E	F	F	D	Q	G	D	R	E	R	K	E	L	N	I	E	P	T	-	D	L	M	N	R	-	-	-	-	-	E	K	K	N	K	I	P	S	M	Q	V	G	F
PDE6A	L	L	V	A	A	E	F	W	E	Q	G	D	L	E	R	T	V	L	Q	Q	N	P	I	-	P	M	M	D	R	-	-	-	-	-	N	K	A	D	E	L	P	K	L	Q	V	G	F
PDE7A1	E	K	V	T	E	E	F	F	H	Q	G	D	I	E	K	-	K	Y	H	L	G	V	S	-	P	L	C	D	R	-	-	-	-	-	H	T	-	E	S	I	A	N	I	Q	I	G	F
PDE8A1	A	R	I	S	E	E	Y	F	S	Q	T	D	E	E	K	-	Q	Q	G	L	P	V	V	M	P	V	F	D	R	-	-	-	-	-	N	T		C	S	I	P	K	S	Q	I	S	F
PDE9A2	D	C	L	L	E	E	Y	F	M	Q	S	D	R	E	K	-	S	E	G	L	P	V	A	-	P	F	M	D	R	-	-	-	-	-	D	K	-	V	T	K	A	T	A	Q	I	G	F
PDE10A2	N	D	I	Y	A	E	F	W	A	E	G	D	E	M	K	-	K	L	G	I	Q	P	I	-	P	M	M	D	R	-	-	-	-	-	D	K	K	D	E	V	P	Q	G	Q	L	G	F
PDE11A2	E	L	V	T	S	E	F	F	E	Q	G	D	R	E	R	L	E	L	K	L	T	P	S	-	A	I	F	D	R	-	-	-	-	-	N	R	K	D	E	L	P	R	L	Q	L	E	W
TbrPDEB1	M	A	V	T	E	E	F	Y	R	Q	G	D	M	E	K	-	E	R	G	V	E	V	L	-	P	M	F	D	R	-	-	-	-	-	S	K	N	M	E	L	A	K	G	Q	I	G	F
LmjPDEB1	M	A	V	T	E	E	F	Y	R	Q	G	D	M	E	K	-	E	K	G	V	E	V	L	-	P	M	F	D	R	-	-	-	-	-	S	K	N	N	E	L	A	R	G	Q	I	G	F
TcrPDEC1	L	V	I	L	Q	E	F	A	D	Q	A	E	D	E	R	-	R	R	G	L	P	V	T	-	P	G	F	E	T	-	-	-	-	-	P	-	-	S	S	V	E	K	S	Q	I	P	F
