# Supplementary material for: The single cyclic nucleotide-specific phosphodiesterase of the intestinal parasite Giardia lamblia represents a potential drug target
Source: PLoS Negl Trop Dis. 2017 Sep 15;11(9):e0005891. doi: 10.1371/journal.pntd.0005891 (PMC5617230; doi:10.1371/journal.pntd.0005891)
Supplement: S1 Table — (PDF) [file pntd.0005891.s006.pdf]

S1 Table. Sequence identity in the catalytic core between GlPDE and other PDEs.

The catalytic core region of different PDEs (helix 3-16) was aligned using the program MSAprobs with standard parameters.
% identity scores were calculated using a script, whereby gaps were excluded.

	substr.spec:	Giardia	Human PDEs												Kinetoplastid PDEs			*Plasmodium falciparum* PDEs			
	cAMP,cGMP	GlPDE	1B	2A	3B	4A	4D	5A	6A	7A	8A	9A	10A	11A4	TbrPDEB1	TcrPDEC1	LmjPDEB1	PfPDEαB	PfPDEβ	PfPDEγ	PfPDEδ
**GlPDE**	A	100	28	23	28	30	32	23	21	27	29	26	22	26	25	20	28	25	24	27	21
**hPDE1B**	A,G	28	100	27	38	40	42	27	28	34	34	30	25	29	33	30	31	30	33	32	28
**hPDE2A**	A,G	23	27	100	29	29	30	37	32	26	26	29	38	39	29	22	30	26	29	27	23
**hPDE3B**	A,G	28	38	29	100	36	37	24	25	32	32	29	23	26	30	31	31	27	29	32	26
**hPDE4A**	A	30	40	29	36	100	89	28	32	36	36	32	26	29	31	31	32	26	28	29	25
**hPDE4D**	A	32	42	30	37	89	100	27	31	36	35	32	27	30	31	30	31	27	29	31	25
**hPDE5A**	G	23	27	37	24	28	27	100	43	29	24	30	43	49	31	22	31	22	25	24	23
**hPDE6A**	G	21	28	32	25	32	31	43	100	27	24	28	36	42	30	20	29	23	24	22	19
**hPDE7A**	A	27	34	26	32	36	36	29	27	100	34	32	27	28	31	25	32	29	28	27	28
**hPDE8A**	A	29	34	26	32	36	35	24	24	34	100	34	21	27	29	28	29	27	28	30	26
**hPDE9A**	G	26	30	29	29	32	32	30	28	32	34	100	27	30	29	29	30	29	31	27	26
**hPDE10A**	A,(G)	22	25	38	23	26	27	43	36	27	21	27	100	38	28	21	29	24	23	24	22
**hPDE11A4**	A,G	26	29	39	26	29	30	49	42	28	27	30	38	100	33	24	33	24	27	25	20
**TbrPDEB1**	A	25	33	29	30	31	31	31	30	31	29	29	28	33	100	25	70	27	26	25	21
**TcrPDEC1**	A,G	20	30	22	31	31	30	22	20	25	28	29	21	24	25	100	22	21	25	25	24
**LmjPDEB1**	A	28	31	30	31	32	31	31	29	32	29	30	29	33	70	22	100	24	26	24	22
**PfPDEαB**	G	25	30	26	27	26	27	22	23	29	27	29	24	24	27	21	24	100	35	32	31
**PfPDEβ**	?	24	33	29	29	28	29	25	24	28	28	31	23	27	26	25	26	35	100	41	35
**PfPDEγ**	?	27	32	27	32	29	31	24	22	27	30	27	24	25	25	25	24	32	41	100	30
**PfPDEδ**	G,(A?)	21	28	23	26	25	25	23	19	28	26	26	22	20	21	24	22	31	35	30	100

Summary:

**% identitiy between GlPDE and hPDEs:**			**% identity between human PDEs:**		
min =	21	hPDE6A	min =	21	hPDE8 - hPDE10
max =	32	hPDE4D	max =	49	hPDE5 - hPDE11
average =	26		average =	31	

color coding:

0	25	50	75	100
