# Supplementary material for: The single cyclic nucleotide-specific phosphodiesterase of the intestinal parasite Giardia lamblia represents a potential drug target
Source: PLoS Negl Trop Dis. 2017 Sep 15;11(9):e0005891. doi: 10.1371/journal.pntd.0005891 (PMC5617230; doi:10.1371/journal.pntd.0005891)
Supplement: S1 File — (PDF) [file pntd.0005891.s007.pdf]

S1 File. Alignment of the GlPDE homologs from all five sequenced *G.lamblia* isolates

```
CLUSTAL O(1.2.1) multiple sequence alignment

                   >>>>>>>>>>>>>>>>>>>>>>>>>>>>>>mito>>>>>>>>>>>>>>>>>>>>>>>>>>
GL50803_14058      --------------MISLCLAAGSLLLASSILVRLCSHSFIVNVHNLSTMILGLFSLLFL     46
DHA2_150867        MCHKQGKKRRAYRRMISLCLAAGSLLLASSILVRLCSHSFIVNVHNLSTMILGLFSLLFL     60
GLP15_4333         --------------MISLCLAAGSLLLAGSILIRLCSHSFIVNVHNLSTMILGLFSLLFL     46
GL50581_303        --------------MISLCLAAGSLFLVGSILIRLYFHSFIADIQNISTMVLGFFSLLFL     46
GSB_153349         --------------MISLCLAAGSLFLVGSILIRLYFHSFIADIQNISTMVLGFFSLLFL     46
                                 ***********:*..***:**  ****.:::*:***:**:******

                   >>>>>>>>>>>>>>>>>>>>>>
GL50803_14058      TGFLFLSTDIHIYLGISVITAGRATSILACLHLLSFWCLIIVTFQTRLYFLLLSSTLKLL     106
DHA2_150867        TGFLFLSTDIHIYLGISVITAGRATSILACLHLLSFWCLIIVTFQTRLYFLLLSSTLKLL     120
GLP15_4333         TGFLFLSTGIHIYLGISVIVAGRAVSILACLHLLSFWCLITVTFQTRLYFLLLSSTLKLL     106
GL50581_303        TGSLFLSTGIHEYLGISVIAACRAVSILISLHILAFWCLTLVTFQTGLYFLLLSSTLKLL     106
GSB_153349         TGSLFLSTGIHEYLGISVIAACRAVSILISLHILAFWCLTLVTFQTGLYFLLLSSTLKLL     106
                   ** ***** ** *******.* **.*** .**:*:****  ***** *************

GL50803_14058      PIVTLFGLMTPILLLSVGVVSVLDSMFRFMWVPFFLILCIETANYFLFKYPDRIMWAIMQ     166
DHA2_150867        PIVTLFGLMTPILLLSVGVVSVLDSMFRFMWVPFFLILCIETANYFLFKYPDRIMWAIMQ     180
GLP15_4333         PIVTLFGLMAPILLISIGVVSVLDSMFRFTWVPFFLILCTETANYFLFKYPDRIMWAIMQ     166
GL50581_303        PIVTLFGMVAPILVFSLDTVSILDNMFRFMWVPFLLILCTEIANYLLFKYPDRILWAIMQ     166
GSB_153349         PIVTLFGMVAPILVFSLDTVSILDNMFRFMWVPFLLILCTEIANYLLFKYPDRILWAIMQ     166
                   *******:::***::*: .**:**.**** ****:**** * ***:********:*****

GL50803_14058      RIVRTTSLQTSSERLSVVALLLPPEAGKECLLESGSESGIQRSAAGGLSGLNTRPTHVKL     226
DHA2_150867        RIVRTTSLQTSSERLSVVALLLPPEAGKECLLESGSESGIQRSAAGGLSGLNTRPTHVKL     240
GLP15_4333         KIVRTTSLQTSSERLSVVALLLPPETGKECLLESGSESDIQRAATRGLSNLATSHTHVRL     226
GL50581_303        RIVRTTAMQTSSEKLSVIAPLLPPDIHKVRS----SESSTPRDSAEGSSDSSALSAPVKL     222
GSB_153349         RIVRTTAMQTSSEKLSVIAPLLPPDIHKVRS----SESSTPRDSAEGSSDSSALSAPVKL     222
                   :*****::*****:***:* ****:  *       ***   * :: * *   :  : *:*

GL50803_14058      KKTIVDGLPDYSPVAADCEPTYQSVWKQLNESINLNAPTTRSSFAAIISTIKKHRFAEAP     286
DHA2_150867        KKTIVDGLPDYSPVAADCEPTYQSVWKQLNESINLNAPTTRSSFAAIISTIKKHRFAEAP     300
GLP15_4333         KKTILDDLPDYSSVITNYEPTYQSVQKQLDESINLNTPTTRSSFAAIVSTIRKHRFAESP     286
GL50581_303        KKTVLNGLLNYSSITANQPTTCQGTQKQLDDFIDLKASNTRTSLAAIISTIKRHHFAEPP     282
GSB_153349         KKTVLNGLLNYSSITANQPTTCQGTQKQLDDFIDLKASNTRTSLAAIISTIKRHHFAEPP     282
                   ***::: * :** : ::   * *.. ***:: *:*:: .**:*:***:***::*:*** *

GL50803_14058      PINFFPHIEPTMFYLVTVSLLFVTALGLNVCYKYLLRGKLMEILPQLIHFTHKSTESEYM     346
DHA2_150867        PINFFPHIEPTMFYLVTVSLLFVTTLGLNVCYKYLLRGKLMEILPQLIRFTHKSTESEYM     360
GLP15_4333         PINFFPHIEPTMFYLVLVSLLFIIALSLNICYKYLLRERLMGLLPQLIRFTHRSIEPDHM     346
GL50581_303        PINFLPHIEPTAFYLVTVSMLFVTALGLSISYNYLLRDKLMEIHPQLIRFVRGSPVPDSI     342
GSB_153349         PINFLPHIEPTAFYLVTVSMLFVTALGLSISYNYLLRDKLMEIHPQLIRFVRGSPVPDSI     342
                   ****:****** **** **:**: :*.*.:.*:**** :** : ****:*.: *   : :

GL50803_14058      SSSASIYATFLEFINNYNIFPAMHAILILQMIFIVPLCNGNTVDVMMVFTNPVIFQVFAR     406
DHA2_150867        SSSASIYAAFLEFINNYSIFPTMHAILILQMIFIVPLCNGNTVDVMMVFTNPVIFQVFAR     420
GLP15_4333         TSSASIYTAFLELINNYSAFPAMHAILILQMIFIVPLCNGNAVDVMMVFTNPVIFQVFAR     406
GL50581_303        STSTHIYTAFLNLINKYSIFPAMHAVLILQMIFMVPLCNSNTVDVMTVFTNPVIFQVFAR     402
GSB_153349         STSTHIYTAFLNLINKYSIFPAMHAVLILQMIFMVPLCNSNTVDVMTVFTNPVIFQVFAR     402
                   ::*: **::**::**:*. **:***:*******:*****.*:**** *************
```

```
GL50803_14058     WLESNRFGAPLAFLIDCYFYRQRPSYERMIVNKIYVKWIDMLANGMLPCTGIALSEVEFL     466
DHA2_150867       WLESNRFGAPLAFLIDCYFYRQRPSYERMIVNKIYVKWIDMLANGMLPCTGIALSEVEFL     480
GLP15_4333        WLESNRFGAPLAFLIDCYFYRQRPSYERMIVNKIYVKWIDMLANGMLPCTGVALSEVEFL     466
GL50581_303       WLESNRFGAPLAFLIDCYLYRQRPSYETVTINKIYVKWIDMLANGMIPCTGAALSEVEFL     462
GSB_153349        WLESNRFGAPLAFLIDCYLYRQRPSYETVTINKIYVKWIDMLANGMIPCTGAALSEVEFL     462
                  ******************:******** : :***************:**** ********

GL50803_14058     VSRVMSAGLNKHYFNDMTSCDNMLSILDSFGTTSSEKLLSKIRGKRGPTGVSSAFCSTNT     526
DHA2_150867       VSRVMSAGLNKHYFNDMTSCDNMLSILDSFGTTSSEKLLSKIRGKRGPTGVSSAFCSTNT     540
GLP15_4333        VSRVMSAGLNKHYFNDMTSCDNMLSILDSFGTTSSDKLLSKIRGKRGPAGVSSIFCSANT     526
GL50581_303       VSRVMSAGLKKHHFNDMTPYDNMLSILDSFGTTTSDKLLSKMRNKRSPAGVSSAFCSSSA     522
GSB_153349        VSRVMSAGLKKHHFNDMTPYDNMLSILDSFGTTTSDKLLSKMRNKRSPAGVSSAFCSSSA     522
                  *********:**:*****  *************:*:*****:* **.*:**** ***:.:
                     ---——end of TMH region in GL50803_14058————┐
GL50803_14058     SNYHLNRSLSTDFVINMDVFTAIEIELSIIVYICAIKFVYSLAPIYFRTLAYLSTLENHA     586
DHA2_150867       SNYHLNRSLSTDFVINMDVFTAIEIELSIIVYICAIKFVYSLAPIYFRTLAYLSTLENHA     600
GLP15_4333        SNHQLNRSLSTDFVINMDVFTAIEIELSIVVYICAIKFLYSLAPIYFRTLTYLSTLESHA     586
GL50581_303       STHQQNRSLSTDFIINMDVFTAIEIELSIVVYICAIKFLYSLSPIYFRTLTYLSTLEISA     582
GSB_153349        STHQQNRSLSTDFIINMDVFTAIEIELSIVVYICAIKFLYSLSPIYFRTLTYLSTLEISA     582
                  *.:: ********:***************:********:***:*******:******  *

GL50803_14058     TMPCVLFRLDNMERQGLMKGVFSNHITVSDPFLPKGGEKALSKGVSSKSYCNIYLKMLFK     646
DHA2_150867       TMPCVLFRLDNMERQGLMKGVFSNHITVSDPFLPKGGEKALSKGVSSKSYCNIYLKMLFK     660
GLP15_4333        TMPCVLFRLDNMERQGLMKGVLSNHVTVCDPFLSKGGENDLSTGVSSKSYCNIYLKMLFK     646
GL50581_303       TMPYVLFRLDNMEHQRLAKGVFANYAITHDPFLSKGSEEGLNRGVSSKNYCDIYLKMLFK     642
GSB_153349        TMPYVLFRLDNMEHQRLAKGVFANYAITHDPFLSKGSEEGLNRGVSSKNYCDIYLKMLFK     642
                  *** *********:* * ***::*:  . **** **.*: *. *****.**:********

GL50803_14058     SLCNYAEADLRLELTLLAVLNKCSPNILFSAVTPRTIAKLSEAHKKSLLLDQLTLLSNIS     706
DHA2_150867       SLCNYAEADLRLELTLLAVLNKCSPNILFSAVTPRTIAKLSEAHKKSLLLDQLTLLSNIS     720
GLP15_4333        SLQNYAEADLRLELTLLAILNKCSPNILFSAVTPRTITKLSEAHKKSLLLDQLTLLSNIS     706
GL50581_303       TLQGCRETDLRPELTLLAVLNTCSPNILFSTMTPKTIGKLSEAQKKSLLLDQLTLLSNIS     702
GSB_153349        TLQGCRETDLRPELTLLAVLNTCSPNILFSTMTPKTIGKLSEAQKKSLLLDQLTLLSNIS     702
                  :*    *:*** ******:**.********::**:** *****:****************

GL50803_14058     AYNMNLDPRQKLGSTLLASQNLILKNLSHTRARRNSLIGPKILSSGKRGAASEAGEGTKD     766
DHA2_150867       AYNMNLDPRQKLGSTLLASQNLILKNLSHTRARRNSLIGPKILSSGKRGAASEAGEGTKD     780
GLP15_4333        VYNMSLDSKQQSGSTLLASQNLILKNLAHTRARRNSLIGPKTLSSRKKDTTLGTSENTKD     766
GL50581_303       TYNMNLDLKQSSSSTLIASQNLILKNLSHTRARRNSLIGPKILSSRRRLIASETDRSAKA     762
GSB_153349        TYNMNLDLKQSSSSTLIASQNLILKNLSHTRARRNSLIGPKILSSRRRLIASETDRSAKA     762
                  .***.** :*. .***:**********:************* *** ::  :  : . :*

GL50803_14058     KSHIPDSTLFSYFDSSEEKLVTLLKEILRTRRLDTAMQSRILTVCQLLDPDLHVSSAILQ     826
DHA2_150867       KSRIPDSTLFSYFDSSEEKLVTLLKEILRTRRLDTAMQSRILTVCQLLDPDLHVNSAILQ     840
GLP15_4333        RSHITDNTLFSYFDSSEEKLVTLLKEILRTRKLDTAMRSRVLTVCQLLDPDLRIHSAILQ     826
GL50581_303       KSPINDNVLFSYFDSSEEKLVTLLKEILRSKDLDNAMRSRILAVCQLLDPNSHIHSAILQ     822
GSB_153349        KSPINDNVLFSYFDSSEEKLVTLLKEILRSKDLDNAMRSRILAVCQLLDPNSHIHSAILQ     822
                  :* * *..*********************:: **.**:**:*:*******: :: *****

GL50803_14058     DSKDPSAKSEDWGKLLNDIKGMKLMNLTVDDKSEISEQLRTLDEGRYEIERPQSTRTVLS     886
DHA2_150867       DSKDPSAKPEDWGKLLNDIKGMKLMNLTVDDKSEISEQLRALDEGRYEIERPQSTRTVLS     900
GLP15_4333        DSKDASTKSEDWGKLLNDIKGMKLMSLTINDKSEINEQLRALDEGRYEIERPQNMRTVLS     886
GL50581_303       DGNAPAAETDDWGKLLNDIRGMKLMNLVISDRSEFGEQLRSLDEGRYELAGPPKKQTVLS     882
GSB_153349        DGNAPAAETDDWGKLLNDIRGMKLMNLVISDRSEFGEQLRSLDEGRYELAGPPKKQTVLS     882
                  *.:  ::: :*********:*****.*.:.*:**: ****:*******:  * . :****

GL50803_14058     YSSSTNISKQSVENMLQLYSKRYPEHIRMPDVSQREFRVNNTYYRLDQSGELVLTLQQRI     946
DHA2_150867       YSSSTNISKQSVENMLQLYSKRYPEHIRMPDVSQREFRVNNTYYRLDQSGELVLTLQQRI     960
GLP15_4333        YSSSTSISRQSVEDMLQLYSKRYPEHIRMPDVSQREFRVNNTYYRLDQSGELVLTLQQRI     946
GL50581_303       YNSSTSISKQSVEDMLQLYSKQYPEHIRMPDSSQREFRVNNTYYRLNQSGELILTLQQRI     942
GSB_153349        YNSSTSISKQSVEDMLQLYSKQYPEHIRMPDSSQREFRVNNTYYRLNQSGELILTLQQRI     942
                  *.***.**:****:*******:********* **************:*****:*******
```

```
                                                                     |==CAT DOMAIN=>
                                                                     +----H1---
GL50803_14058      LQAVLNIAVIRTLEYFETGDESAMTLTVTSELAEKYGVDCSNIKLAIEYNLDKISKYICQ     1006
DHA2_150867        LQAVLNIAVIRTLEYFETGDESAMTLTVTSELAEKYGVDCSNIKLTVEYNLDKISKYICQ     1020
GLP15_4333         LQAVLNIAVIRTLEYFETGDENAMTLTVTSELAEKYRVDCSNIKLAVEYNLDKISKYICQ     1006
GL50581_303        LQAVLNITIIRTLEYFETGDESAITLTVTSDLADKYGLDCSNIKLAIEYNLDKISRYVCQ     1002
GSB_153349         LQAVLNITIIRTLEYFETGDESAITLTVTSDLADKYGLDCSNIKLAIEYNLDKISRYVCQ     1002
                   *******::************.*:******:**:** :*******::********:*:**

                   +       +--H2-+    +----H3----+   +H4    +----H5-----+
GL50803_14058      NIESSSFDVSAMNTYTRTYGLTAVGYILAKLLGITTYFSIHDNVLLAVLIELESSYTSTL     1066
DHA2_150867        NIESSSFDVSAMNTYTRTYGLTAVGYILAKLLGITTYFSIHDNVLLAVLIELESSYTSTL     1080
GLP15_4333         NIESSSFDVNTINTYTRTYGLTAVGYILAKLLGITTYFSIHDNVLLAVLIELESSYTSTL     1066
GL50581_303        NIESPSFDVSAMNAYTRTYGFTAIGYILAKILGITTYFSIHDNVLLAVLIELESSYTSTL     1062
GSB_153349         NIESPSFDVSAMNAYTRTYGFTAIGYILAKILGITTYFSIHDNVLLAVLIELESSYTSTL     1062
                   **** ****.::*:******:**:******:*****************************

                   ●● +-●----H6------+ |~~~~~~~~~~35-aa insert~~~~~~~~~~~|
GL50803_14058      YHNKLHAADVAQMSMYMLSTVYCSLISESPKHPFLCVYKAMRQYKESDYSRLITEQPRQA     1126
DHA2_150867        YHNKLHAADVAQMSMYMLSTVYCSLISESPKHPFLCVYKAMRQYKESDYSRLITEQPRQA     1140
GLP15_4333         YHNKLHAADVAQMSMYMLSNVYCSLINESPKHPFLCVHKAMLRYKESDYSRLIAEQPRQA     1126
GL50581_303        YHNKLHAADVAHMSMYMLSTVYCSLLDESPKHPFLYVYKAMQQYNENDYDRLVEEQPKRA     1122
GSB_153349         YHNKLHAADVAHMSMYMLSTVYCSLLDESPKHPFLYVYKAMQQYNENDYDRLVEEQPKRA     1122
                   ***********:*******.*****:.******** *:*** :*:*.**.**: ***::*

                                          |<=======H-Loop======>|
                      +-----H7----+●●  ●     +-H8-+ +-H9-+     +-----H10-----+
GL50803_14058      LIRPVDFLALLFGSLCHDLGHTGIDNLFCINTENALALLYNDEAPLEHAHATLSWHIITQ     1186
DHA2_150867        LIRPVDFLALLFGSLCHDLGHTGIDNLFCINTENALALLYNDEAPLEHAHATLSWHIITQ     1200
GLP15_4333         LIRPVDFLALLFGSLCHDLGHTGIDNLFCINTENALALLYNDEAPLEHAHATLSWHILTQ     1186
GL50581_303        LIRPVDFLALLFGSLCHDLGHTGIDNLFCINTENALALLYNDEAPLEHAHATLSWHIITQ     1182
GSB_153349         LIRPVDFLALLFGSLCHDLGHTGIDNLFCINTENALALLYNDEAPLEHAHATLSWHIITQ     1182
                   *********************************************************:**

                            +------H11-----+   ●------H12-----+             +---
GL50803_14058      MAVYFKHFTPCQYREFRALFLEIILATDMSTHFNFLRRLESLDEDLIIKILEHHNDSEIA     1246
DHA2_150867        MAVYFKHFTPCQYREFRALFLEIILATDMSTHFNFLRRLESLDEDLIIKILEHHNDSEIA     1260
GLP15_4333         MAIYFKHFTPGQYREFRALFLEIILATDMSTHFNFLRRLESISKDIIVKILEHHNDSEIA     1246
GL50581_303        MAIYFKHFTSSQYREFRALFLEIILATDMSTHFSFLRRLESFDKSFISKIFERHNTSEIS     1242
GSB_153349         MAIYFKHFTSSQYREFRALFLEIILATDMSTHFSFLRRLESFDKSFISKIFERHNTSEIS     1242
                   **:******  *********************.*******:.:.:* **:*:** ***:

                                                   %     |<========M-Loop======
                   ---H13--------●● ●●   +--●---●--●●--●--H14---+      ●
GL50803_14058      LLRWYILKVCIKFGDLSNPCRPIEISTRYAVALMNEFWSLGDLMLECGLEPDKIKTRPQK     1306
DHA2_150867        LLRWYILKVCIKFGDLSNPCRPIEISTRYAVALMNEFWSLGDLMLECGLEPDKIKTRPQK     1320
GLP15_4333         LLRWYILKVCIKFGDLSNPCRPIEISTRYAVALMNEFWSLGDLMLECGLEPDKIKTRPQK     1306
GL50581_303        LLRWYILKVCIKFGDLSNPCRPIEISTRYAVALMNEFWSLGDLMLECGLEPDKIKTRPQK     1302
GSB_153349         LLRWYILKVCIKFGDLSNPCRPIEISTRYAVALMNEFWSLGDLMLECGLEPDKIKTRPQK     1302
                   ************************************************************

                             @  %
                   ===>|  +-●●--●-----H15------+        +-------H16---●--------
GL50803_14058      GEESLIIANSQIGFTQSIVKGFWTVVERFWKALAGVEFSDLQANLNATVEHWQNVRSEIE     1366
DHA2_150867        GEESLIIANSQIGFTQSIVKGFWTVVERFWKALAGIEFSDLQANLNATVEHWQNVRSEIE     1380
GLP15_4333         GEESLIIANLQIGFTQNIVKGFWTAVERFWKALADVEFKDLQANLNATVEHWQHVRSEIE     1366
GL50581_303        GEESLIIANSQIGFTQSIVKGFLIVVESFWKALADVEFQDLQENLSATVEHWQNIRAEIE     1362
GSB_153349         GEESLIIANSQIGFTQSIVKGFLIVVESFWKALADVEFQDLQENLSATVEHWQNIRAEIE     1362
                   ********* ******.*****  .** ****** :**.*** **.*******.:*:***

                   -+
GL50803_14058      LDKKE  1371
DHA2_150867        LDKKE  1385
GLP15_4333         LDKKE  1371
GL50581_303        VDKKD  1367
GSB_153349         VDKKD  1367
                   :***:
```

LEGEND

GL50803_14058 : Giardia Assemblage A isolate WB
DHA2_150867 : Giardia Assemblage A isolate DH
GLP15_4333 : Giardia Assemblage E isolate P15
GL50581_303 : Giardia Assemblage B isolate GS
GSB_153349 : Giardia Assemblage B isolate GS_B

Humans are infected by assemblages A and B.

<u>Matches in the alignment (shown below the sequences):</u>

* = all residues in that column are identical
: = conserved substitutions
. = semi-conserved substitutions

<u>Sequence annotations (shown above the sequences):</u>

`>>>>mito>>>>` = predicted as mitochondrial targeting signal by the program MitoProt II
`|==CAT=====>` = putative start of the catalytic domain
`<----Hx---->` = alpha helix number x in the catalytic domain (sequence highlighted in blue)
`<==========>` = loops involved in forming or lining the catalytic pocket (H-loop, M-loop)
`|~~~~~~~~~~|` = probable location of the GlPDE-specific 35 amino acid insert
(location was determined by homology modeling using hPDE4D as template)
• = residue of the substrate-binding pocket
@ = invariant glutamine
% = P-clamp residue
